# Study protocol: Strengthening understanding of effective adherence strategies for first-line and second-line antiretroviral therapy (ART) in selected rural and urban communities in South Africa

**DOI:** 10.1371/journal.pone.0261107

**Published:** 2021-12-21

**Authors:** Siphamandla Bonga Gumede, John Benjamin Frank de Wit, Willem Daniel Francois Venter, Samanta Tresha Lalla-Edward

**Affiliations:** 1 Ezintsha, Faculty of Health Sciences, University of Witwatersrand, Johannesburg, South Africa; 2 Faculty of Social and Behavioural Sciences, Department of Interdisciplinary Social Science, Utrecht University, The Netherlands; Nnamdi Azikiwe University, NIGERIA

## Abstract

Multiple factors make adherence to antiretroviral therapy (ART) a complex process. This study aims to describe the barriers and facilitators to adherence for patients receiving first-line and second-line ART, identify different adherence strategies utilized and make recommendations for an improved adherence strategy. This mixed method parallel convergent study will be conducted in seven high volume public health facilities in Gauteng and one in Limpopo province in South Africa. The study consists of four phases; a retrospective secondary data analysis of a large cohort of patients on ART (using TIER.Net, an ART patient and data management system for recording and monitoring patients on ART and tuberculosis (TB)) from seven Johannesburg inner-city public health facilities (Gauteng province); a secondary data analysis of the Intensified Treatment Monitoring Accumulation (ITREMA) trial (a randomized control trial which ran from June 2015 to January 2019) conducted at the Ndlovu Medical Center (Limpopo province); in-depth interviews with people living with Human Immunodeficiency Virus (PLHIV) who are taking ART (in both urban and rural settings); and a systematic review of the impact of treatment adherence interventions for chronic conditions in sub-Saharan Africa. Data will be collected on demographics, socio-economic status, treatment support, retention in care status, disclosure, stigma, clinical markers (CD4 count and viral load (VL)), self-reported adherence information, intrapersonal, and interpersonal factors, community networks, and policy level factors. The systematic review will follow the Preferred Reporting Items for Systematic Reviews and Meta-Analysis (PRISMA) reporting and Population, Interventions, Comparisons and Outcomes (PICO) criteria. Analyses will involve tests of association (Chi-square and t-test), thematic analysis (deductive and inductive approaches) and network meta-analysis. Using an integrated multilevel socio-ecological framework this study will describe the factors associated with adherence for PLHIV who are taking first-line or second-line ART. Implementing evidence-based adherence approaches, when taken up, will improve patient’s overall health outcomes. Our study results will provide guidance regarding context-specific intervention strategies to improve ART adherence.

## Introduction

Inconsistent adherence to treatment is a contributing factor to poor health outcomes of people affected by numerous health conditions, including HIV, tuberculosis, diabetes mellitus (DM) and hypertension [1–3]. The World Health Organization (WHO) defines adherence as the degree to which a patient is able to follow a treatment schedule and take medication at recommended times [4–6]. In the context of HIV, lapses in adherence to medication can lead to the development of viral rebound, which can result in immunosuppression and viral resistance [4,7–9].

WHO recommends the use of the HIV drug dolutegravir (DTG) as the preferred first-line and second-line ART treatment regimens for all populations due to its formidable resistance barrier and improved tolerability [10–12]. Despite the advantages of this first-line regimen, in South Africa between 20%-30% of patients with HIV experience clinical, immunological or virological failure from first-line ART due to lapses in adherence [13–15]. This is a concern because of the clinical and cost implications attached to treatment failure [16,17].

Adherence to ART is a complex process that is affected by multiple factors, and numerous studies have attempted to establish what the barriers and facilitators of ART adherence are [18–20]. Individual-level factors such as age, sex, ethnicity, HIV status disclosure and forgetfulness, have been reported as important in predicting ART adherence [21]. However, using individual-level factors one is only able to report a limited proportion of the variability in non-adherence [22]. Good interpersonal relationships between patients and care givers or treatment supporters including healthcare providers, an intimate partner, family members, and friends have been reported as predictors for good adherence [22,23]. In contrast to intrapersonal and interpersonal factors, the community level factors such as poverty, HIV related stigma and discrimination against patients on ART introduce barriers to ART adherence [24]. Additional to community level factors, awareness of healthcare policy level factors like HIV treatment guidelines, policies, and best practices are imperative in ensuring good adherence and maintenance of the continuum of care [25].

### Conceptual framework

Various studies have demonstrated that there are many factors that play an important role in maintaining adherence behaviour [26–29]. These factors have been explored using several models, including: 1) Anderson’s Health Care Utilisation model [30], which is a framework that considers predisposing factors (individual’ own personality and behaviour), enabling factors (patient and health provider relationship, community education) and need factors (patient’s beliefs, alternative medicine treatment options, community support); 2) the Dahlgren-Whitehead ’rainbow model’ [31], a model that builds the relationship between the individual, the environment they live in and health; 3) Information-Motivation-Behavioural skills model (IMB model) [32–34], a model that views adherence behaviour as a function of the interrelations between adherence-related information, motivation, and behavioural skills; 4) the socio-ecological conceptual framework [35,36], which takes into consideration the individual, and their connections to other people, and how they adapt their behaviour to the social environment. This model suggests that an individual’s behaviour is cohesive in a dynamic network of intrapersonal, interpersonal characteristics, community features and existing health policies [35,36].

**Fig 1:**
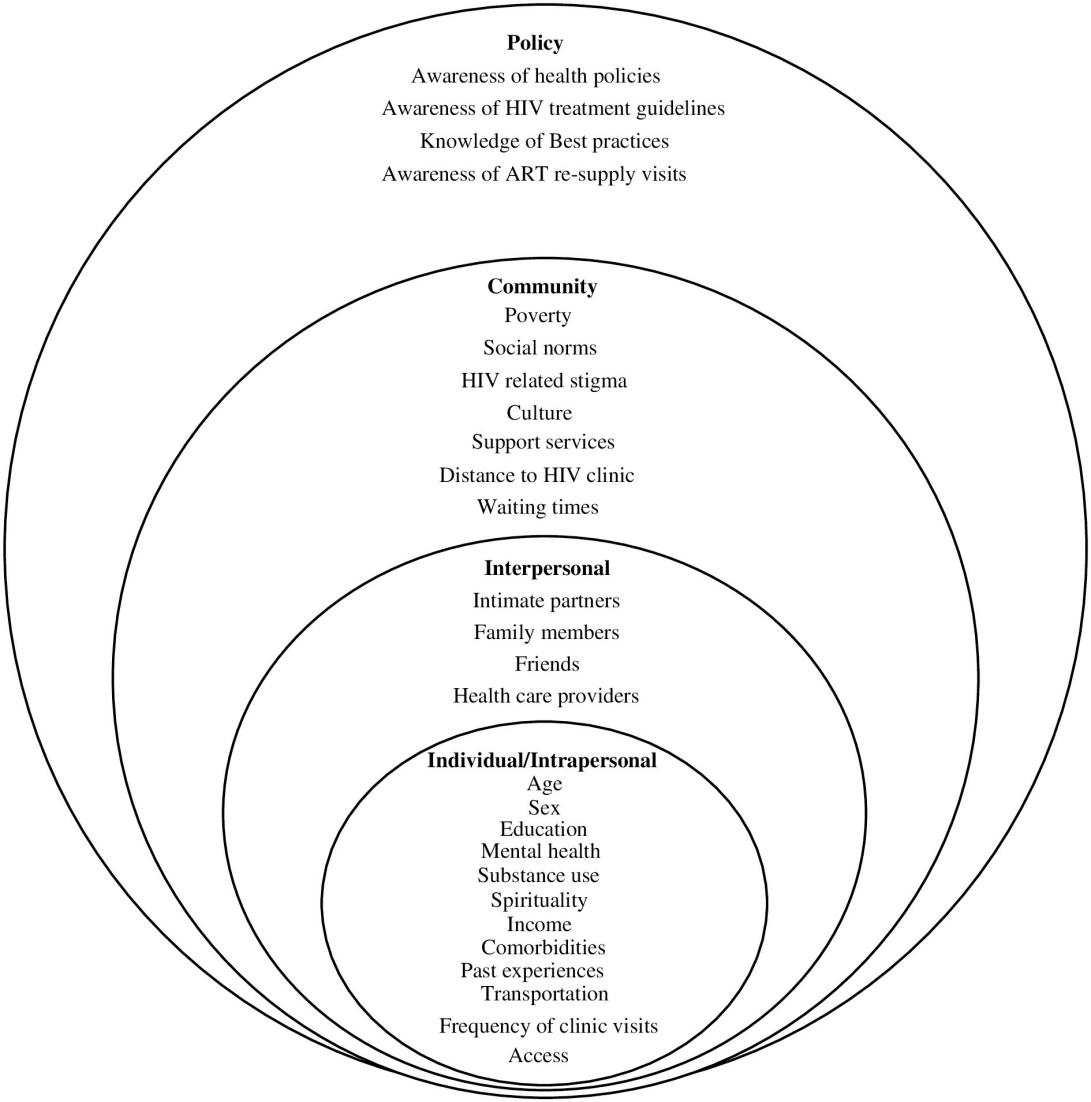
An adapted socio-ecological framework. An adapted socio-ecological framework that depicts the layers of individual, relationship, community, and healthcare policy level factors which influence the processes of treatment adherence and retention in care [36].

Our study will adapt the socio-ecological conceptual framework to investigate multilevel and interactive factors such as individual/intrapersonal, interpersonal, community, and health policy level factors that affect adherence to ART (Fig 1). It will explore the different aspects of these factors that act either as barriers or facilitators of adherence to ART.

#### Intrapersonal level factors

The intrapersonal level of the socio-ecological conceptual framework comprises individual knowledge, attitudes, beliefs, perceptions, and skills that influence behaviour [35]. It further includes age, sex, income, mental health, education, substance use, spirituality, comorbidities and past experiences [36].

#### Interpersonal level factors

Here the participant’s social network or relationships with other people including family, friends, peers, intimate partner and health care providers [35] are included. This level also highlights trust and communication as factors that builds a relationship between patients and care givers or treatment supporters [25].

#### Community level factors

The components of the community-level factors that may influence patient’s adherence to ART incorporate cultural views and social norms towards ART access, HIV related stigma, poverty, and available support services within the community [36].

#### Policy level factors

The public policy level is shaped by local, and government laws regarding access and adherence to ART. The socio-ecological conceptual framework reports on awareness of macro level factors which includes health policies, HIV treatment guidelines and best practices, [22,36].

### Research gap

Although ample literature on adherence to ART has been available for a while, there remains a dearth of studies relating to the multi-level factors associated with adherence to treatment and processes shaping adherence behaviour, particularly in South Africa. This lends to the lack of understanding of the interplay between the various factors involved at different levels of the treatment taking behaviour of the ART patient.

In South Africa specifically, there is lack of reported knowledge about the effectiveness and impact of strategies currently employed to promote adherence in people living with HIV (PLHIV) who are taking ART (particularly young patients, males, and those experiencing severe treatment related side effects) [37,38]. Unfortunately, there are no quantitative studies of combination ART in South Africa (and even sub-Saharan Africa more generally) that have used a social-ecological perspective or approach. This is despite the understanding that patients on ART require comprehensive adherence strategies and strict viral load monitoring [39,40].

Until 2017, there were minimal related treatment adherence strategies (routine blood monitoring, targeted adherence counselling, and adherence clubs [41–43]) employed by the South African government [41]. However, effectiveness of these interventions have not been widely reported on. Additionally, the patients’ perspectives seem to be given little consideration in the adherence intervention development and implementation. It is therefore important to assess effectiveness of existing strategies from the perspectives of PLHIV taking ART in order to recommend and develop relevant and acceptable strategies to patients [44,45]. This is evidenced in a study conducted in Malawi which highlighted how critical it is for health programmers to prioritize adherence view points from patients as part of strengthening the clinical monitoring strategy used [46].

### Rationale for the study

Consistent high levels of adherence to HIV medication are important for viral suppression, consequently preventing resistance to ART and progression of the illness [47]. According to the socio-ecological framework, numerous levels of factors affects patients’ adherence to treatment [25]. Therefore, a multilevel socio-ecological framework will provide information about the influence of particular risk factors relative to others, or if combined effects of risk factors are additive or related. The socio-ecological framework will serve as the conceptual framework in our study in order to understand factors affecting treatment adherence at different levels and also guide strategies to improve ART adherence.

#### Aim

To describe the barriers and facilitators to adherence for patients receiving first-line and second-line ART and different adherence strategies utilised.

#### Overarching hypothesis

A socio-ecological framework combined with multi-model data collection will identify the barriers and facilitators to ART adherence and retention in care for PLHIV needed to strengthen adherence interventions.

### Research questions and hypotheses

1. What are the demographic characteristics and clinical indicators associated with virological failure and being lost to follow-up (LTFU) in PLHIV taking first-line and second-line ART in urban communities?
  Hypothesis: Demographic and clinical characteristics are associated with virological failure and being LTFU in patients on ART from complex, and highly mobile urban communities.
2. What are the intra-and inter-personal factors (demographic, socio-economic characteristics), social and community level factors (poverty, social norms, HIV related stigma, culture), structural factors (health systems, support services) and clinical indicators associated with self reported adherence, pill count and virological failure to first-line and second-line ART in rural communities?
  Hypothesis: Individual and community level factors are associated with self-reported non-adherence, suboptimal pill count, and virological failure in patients on ART in the rural setting.
3. What are the perspectives of virally suppressed and unsuppressed first-line and second-line ART patients about treatment adherence in selected urban and rural communities?
  - Are there any differences between people receiving first-line and second-line ART?
  - Are there any differences in treatment taking behaviours between virally suppressed and unsuppressed ART patients?
  - What do virally suppressed and unsuppressed ART patients recommend as adherence strategies?
    Hypothesis: In South African communities, treatment taking behaviour, perceptions of adherence, and recommendations for adherence interventions differ between PLHIV who are virally unsuppressed and those who are virally suppressed.
4. What treatment adherence strategies and interventions have been implemented and evaluated in sub-Saharan Africa for HIV, hypertension, and DM?
  Hypothesis: There are no differences in the effectiveness of adherence interventions implemented in sub-Saharan Africa for HIV, hypertension, and DM.

## Materials and methods

### Study design

The study will employ a mixed method parallel convergent approach conducted in four phases (I-IV). Due to the different approaches for each study objective, we present the study methods based on each objective. While the studies will employ their own separate methods, all the results will be converged/collated into a single discussion to provide recommendations for adherence strategies that can be rolled out in South Africa.

### Materials and methods: Phase I

Objective 1: Assess demographic characteristics and clinical indicators associated with virological failure and LTFU in first-line and second-line patients in an urban community.

#### Study design

The study will employ a quantitative retrospective cohort study using secondary data analysis of data on people with HIV taking ART (18 years and older) recorded in the TIER.Net database.

TIER.Net is the monitoring and evaluation system used by the South African Department of Health for ART patient and data management. It comprises limited demographic information and all treatment and laboratory information from the time of commencement on HIV treatment. This is elaborated on in the data collection section.

The South African Department of Health started providing ART in the public health setting on 01 April 2004. From TIER.Net, we will extract a list of all patients who were initiated on ART from 01 April 2004 to 29 February 2020 in the urban setting (city of Johannesburg region F). The cut-off period of 29 February 2020 was chosen to give ART cohorts a minimum of a 12-month follow-up during which a full clinical assessment could be completed as per guidelines.

#### Study setting

This study will be conducted in seven health facilities in the city of Johannesburg region F.

This includes all levels of care

1. **Primary Health Care**: Jeppe Clinic, Malvern Clinic, Rosettenville Clinic, Yeoville Clinic
2. **Community Health Centre**: Hillbrow Community Health Center (HCHC)
3. **Hospitals**: Charlotte Maxeke Johannesburg Academic Hospital (CMJAH) and South Rand Hospital (SRH)

#### Sampling/Sample size

For this study, all records of people with HIV who were ever initiated on ART between 01 April 2004 (the inception of the South African national HIV treatment programme in the public health setting) and 29 February 2020 from the seven public health facilities will be included. Based on the TIER.Net database, about 130 000 adult patients were initiated on first-line and second-line ART in the seven facilities selected in the city of Johannesburg region F.

#### Data collection

Data will be extracted from the TIER.Net database (as an MS Excel export file). TIER.Net captures demographic information such as patient age, sex, facility name and contact details and also HIV specific information such as HIV diagnosis date, ART start date, regimen at baseline, ART visit dates, CD4 count, viral load done and viral load suppression The system also records all treatment related information, including ART switch. All these variables will be extracted (see S1 Appendix study codebook). The data will be exported to STATA 15.1 for data cleaning and analysis. Records with missing data will be excluded in the final analysis. This will be after conducting all necessary data quality checks, verifications, and triangulations with other data sources.

*Outcome*. VL count is categorized into suppressed (<1000 copies/ml) or unsuppressed (**≥**1000 copies/ml) [48,49]. The status on retention in care for patients will be categorized into active in care, LTFU, transferred out, or recorded dead. For this study, LTFU is defined as having missed a scheduled medical appointment by 90 days or more, as defined by the South African Department of Health [50].

#### Data analysis

Data will be coded and analysed using STATA version 15.1. Tests of association (Chi-square and t-test) between outcome variables and selected demographics characteristics and clinic indicators will be conducted. Outcome variables will include viral load detectability and retention outcomes (active in care, transferred-out, lost to follow-up and dead),. Regression analysis, univariate and multivariate analyses between variables will be built for outcome variables to identify independent predictors. Variables such as age, sex, health facility, baseline regimen, ART start date, baseline CD4 count, most recent CD4 count, ART visit dates, and months on ART will be considered as predictor variables or independent variables. Analysis will include survival analysis which will consider different entry time points into the ART program. Subsequently, patients will not be grouped all together but will be followed up in a 12-month interval when measuring the outcomes (virological failure and lost to follow up).

### Materials and methods: Phase II

Objective 2: Assess socio-demographic and psychosocial associated with adherence (self-reported adherence, pill count and virological failure) in patients on first line and second-line ART in a rural community.

#### Study design

This cohort study using secondary data analysis will be conducted as a sub-study of the Intensified Treatment Monitoring Accumulation (ITREMA) study (Clinicaltrials.gov Identifier NCT03357588) [51]. The ITREMA database will be used to extract ART information. ITREMA is an open-label randomised clinical trial evaluating different treatment monitoring strategies for first-line ART, which ran from June 2015 to January 2019 [51]. ITREMA was conducted at the Ndlovu medical centre in Limpopo province, South Africa. The ITREMA study enrolled adult PLHIV and assessed an intensified HIV-treatment monitoring strategy in a randomised comparison with a control group receiving standard-of-care HIV treatment in accordance with the South Africa National Department of Health guidelines

#### Study setting

Patient enrolment for the ITREMA trial was done between June 2015 and August 2017 at the Ndlovu Medical Centre in Elandsdoorn, Limpopo Province, South Africa.

#### Sampling/Sample size

All the records from ITREMA database will be used. There are 501 ART patients in the ITREMA database.

#### Data collection

Data will be extracted from the ITREMA databases. The ITREMA database contains fields for socio-demographics and psychosocial characteristics. The control variables include sex, age, level of education, employment status, sources of income, household members, food security, mental health, HIV self-efficacy and HIV related stigma (see S1 Appendix study codebook and S6 Appendix ITREMA questionnaire). The data will be exported to STATA 15.1 for data cleaning and analysis.

*Outcome*. Self-reported non-adherence will be measured using three items from the ACTG questionnaire [52]: “How often do you have difficulty in taking your medication on time?, with responses given on 4-point scale (All the time, Most of the time, Rarely, Never), “On average how many days per week would you say that you missed at least one dose of your medication?”, with responses given on a 6-point scale (every day, 4–6 days per week, 2–3 days per week, Once a week, Less than once a week, Never), and “When was the last time you missed taking any of your medications?”, with responses also given on a 6-point scale (Past week, 1–2 weeks ago, 2–4 weeks ago, 1–3 months ago, More than 3 months ago, Never) (see S6 Appendix ITREMA questionnaire). Responding ‘never’ to all three questions will be taken to indicate good self-reported adherence. Suboptimal adherence measured using pill count is defined as a pill count <95%. This threshold is aligned with the WHO cut-off, which considers a pill count ≥ 95% as good adherence for patients taking ART [53]. Virological failure will be defined as viremia ≥1000 copies/ml within 96 weeks of follow-up [48,49].

#### Data analysis

Data will be coded and analysed using STATA version 15.1. Tests of association (Chi-square and t-test) between outcome variables and selected socio-demographics and health related characteristics will be conducted. Outcome variables will include (but not limited to) viral load detectability, retention outcomes (active in care, transferred-out, lost to follow-up and dead), side effects and treatment interruptions (stop and restarting treatment). Regression analysis, univariate and multivariate analyses between variables will be built for outcome variables to identify independent predictors. Variables such as age, sex, health facility, beliefs, education, economic status, employment status, religious status, disclosure, and months on ART will be considered as predictor variables or independent variables.

### Materials and methods: Phase III

Objective 3: Understand adherence in first-line and second-line ART patients who are virologically suppressed and those who are not virologically suppressed in selected urban and rural communities.

#### Study design

The study will employ a qualitative study design approach. Active patients from phase I and II (in both urban and rural settings) will be invited to participate in the in-depth interviews (IDIs) to explore factors including (but not limited to) treatment history, current use of ART, treatment regimen, financial/economic factors, risk behaviours (substance use), psychosocial characteristics cultural beliefs, spirituality and), relationship related factors (treatment support), community level factors (societal norms, stigma, discrimination, disclosure), and policy level factors (understanding/awareness of HIV/ART policies and treatment guidelines).

#### Study setting

This study will be conducted in seven health facilities in the city of Johannesburg region F in Gauteng Province and one in Limpopo Province (Ndlovu Medical Centre).

The seven health facilities in the city of Johannesburg include:

1. **Primary Health Care**: Jeppe Clinic, Malvern Clinic, Rosettenville Clinic, Yeoville Clinic
2. **Community Health Centre**: Hillbrow Community Health Center (HCHC)
3. **Hospitals**: Charlotte Maxeke Johannesburg Academic Hospital (CMJAH) and South Rand Hospital (SRH)

#### Sampling/Sample size

In this study, purposive sampling of patients currently taking ART will be employed to ensure maximum variation among the study sample. We will ensure a diverse sample by considering viral load status (suppressed and unsuppressed time on ART, age (18 years and older), sex (both males and females) and education. Sample size will depend on when saturation is reached; we anticipate that a maximum of 60 IDIs will be conducted across both study settings and viral load status groups (suppressed and unsuppressed). An anticipated number of study participants to recruit is 15 per viral load status group in each study setting (making a total of 30 participants in each study setting).

#### Data collection

Patients for this phase will be contacted using the contact information that they provided for their facility records or databases used in phase I and II. IDIs will be conducted following a semi-structured interview guide. The guide will comprise open-ended questions covering treatment history, current use of ART and multilevel factors derived from the socio-ecological framework. This will include individual level factors including treatment-related factors (ART regimen, use of non-ART medication), financial and economic factors risk behaviours (substance use), psychosocial factors (cultural beliefs, spirituality), interpersonal-level factors (relationship between patients and treatment supporters or caregivers, such as intimate partners, family members, friends and health care workers), community level factors (social norms regarding HIV and ART, HIV-related stigma and discrimination, HIV-status disclosure), health-system factors (access to HIV and ART services including adherence counselling), and policy level factors (HIV testing and treatment guidelines and policies). Additional probes will be included for each question to promote sharing of detailed information regarding their perspectives and experiences, and to ensure clarification if required (see S1 Appendix study codebook and S2 Appendix interview guide). All IDIs will be audio recorded and transcribed verbatim. Transcripts will be translated into English.

*Deductive themes*. Deductive themes may include treatment history, current use of ART, treatment regimen, financial/economic factors, risk behaviors (substance use), psychosocial characteristics cultural beliefs, spirituality and), relationship related factors (treatment support), community level factors (societal norms, stigma, discrimination, disclosure), and policy level factors (awareness of HIV/ART policies and treatment guidelines).

#### Data analysis

Transcripts will be imported and analysed using NVIVO. Data coding will be undertaken using deductive (top down) and inductive approaches (bottom up) [54]. A deductive approach is driven by researchers’ analytic interest in the study, reflecting the broad issues addressed in the interview guide. An inductive approach is used to identify the detailed themes related to the overarching issues that can be identified in the data [54]. Thematic analysis will be used, which is a method for identifying, analysing, and reporting patterns (themes) within data [54,55]. Transcripts will be read while noting similar topics that will be grouped into major topics or themes. Data will be analysed as transcripts become available shortly after interviews are conducted. This will ensure the early identification of emerging themes and assist in the identification of data saturation. Analysis of the IDIs will follow the phases of thematic analysis which are familiarization, generating initial codes, searching for themes, reviewing themes and interpretation [54]. Familiarization (getting grounded into the data collected), will be achieved by reading the transcripts and field notes repeatedly. During the process of generating codes, key emerging ideas and words from the familiarization phase will be recorded from which we will search, identify, and review themes, concepts, categories, and sub-categories. This will be done in keeping with socio-ecological framework, views and experiences that persist from the data. Finally, factors that influence adherence to ART will be identified and grouped into main categories. Analysis will be guided by the codebook which will be developed by the study team post familiarization with the data. There will be multiple independent coders to ensure the reliability of the coding.

### Materials and methods: Phase IV

Objective 4: Assess and compare adherence intervention strategies for the chronic conditions of HIV, hypertension and DM which have been tested and implemented in sub-Saharan Africa (Title: Adherence strategies and interventions for selected chronic conditions in sub-Saharan Africa: a systematic review and meta-analysis) (see S4 Appendix Systematic review protocol).

#### Study design

This systematic review will be designed and reported according to the PRISMA [56] (see S5 Appendix PRISMA checklist), following the registered protocol (CRD42019127564) on the international prospective register of systematic reviews, Prospero [57] (see S4 Appendix Systematic review protocol). The study will use PICO criteria as the search strategy tool.

A systematic review on the impact of treatment adherence interventions in chronic conditions (HIV, hypertension, DM) in sub-Saharan Africa will be conducted to provide context to adherence in sub-Saharan Africa. In this region, HIV remains the leading cause of death more especially in the young and middle-aged adults. However, the burden of non-communicable diseases (NCDs), particularly DM and hypertension, has increased rapidly in recent years [58–60].

#### Study setting

All information from sub-Saharan Africa only will be included for the systematic review.

#### Sampling/Sample size

All interventions described as chronic conditions adherence interventions (HIV/ART, hypertension, DM). Inclusion in the systematic review will be dependent on the criteria set out in the systematic review protocol [57] and reported using the PRISMA reporting guidelines.

#### Data collection

A pre-defined data sheet will be developed for data extraction. The tool will include (but not be limited to): reference (author, title), year of publication, setting or location, sample size, intervention description, participants receiving adherence (in case of comparison) (see S1 Appendix study codebook). The form/tool will be tested before conducting the final searches. One reviewer will conduct all the data extraction while a second reviewer will be responsible for data quality assurance on the extraction and also conduct full text review of the included material.

We will search using several electronic databases. These will include PubMed/Medline, Web of Science, Google Scholar, Scopus, and CINAHL. If necessary, we will contact study authors and request more information on individual studies. Citations and bibliographies of records will be reviewed to identify additional relevant material.

The basic search terms included will be:

*“Chronic conditions”* OR *“hypertension”* OR *“high blood pressure”* OR *“blood pressure”* OR *“arterial hypertension”* OR *“mellitus diabetes type I”* OR *“mellitus diabetes type II”* OR *“Diabetes”* OR *“Sugar”* OR *“HIV”* OR *“Antiretroviral Therapy”* OR *“Antiretroviral Treatment”* OR *“ART”* OR *“ART Programs”* OR *“ART Programmes”* AND *“adherence”* OR *“compliance”* AND *“interventions”* OR *“strategies”* OR *“odds ratio”* OR *“risk ratio”* OR *“evaluation”* OR *“impact”* OR *“effectiveness”* OR *“outcome”* AND *“sub-Saharan Africa”* OR *“sub Saharan Africa”* OR *“sub-Saharan African”* OR *“sub Saharan African”* OR *“Africa”* (Table 1).

**Table 1:** Methodological aspect of the systematic review.

Criteria for study inclusion	Components details
Population (P)	Patients with selected chronic conditions (HIV, hypertension, dDM) in sub-Saharan Africa
Intervention (I)	All interventions listed/described as adherence interventions or strategies for the conditions of HIV, hypertension, DM
Comparisons (C)	Standard of care and other adherence interventions reported on in the review
Outcome (O)	The included studies should report any measurement of adherence to chronic conditions—primarily, effects on adherence behaviour and the changes in health outcomes. There is no preferred measurement for reporting; should there be adequate statistical reporting, a meta-analysis will be considered.
Setting	All studies from sub-Saharan Africa only will be considered for the review.
Language	There will be no language restrictions.
Date	There will be no date/time restrictions.
Publication status	All the documented studies will be considered and included for review. This includes peer reviewed (i.e., papers, manuscripts, and abstracts).
Method	The study will be designed and reported according to PRISMA. PICO will be used as a search strategy approach. This study will describe reported adherence programmes and strategies. There will be a focus on behaviour change techniques used or reported changes in process outcomes of adherence programmes and methods of implementation for HIV, hypertension and /or DM.
Search strategy and selection procedure	We will search using several electronic databases. These will include: PubMed/Medline, Scopus, CINAHL, Web of Science and Google Scholar.
Search terms	(chronic conditions OR hypertension OR high blood pressure OR blood pressure OR arterial hypertension OR mellitus diabetes type I OR mellitus diabetes type II OR Diabetes OR Sugar OR HIV OR Antiretroviral Therapy OR Antiretroviral Treatment OR ART OR ART Programs OR ART Programmes) AND (adherence OR compliance) AND (interventions OR strategies OR odds ratio OR risk ratio or evaluation OR impact OR effectiveness OR outcome) AND (sub-Saharan Africa OR sub Saharan Africa OR sub-Saharan African OR sub Saharan African OR Africa)

The search terms will be adjusted to suit the database being searched. An inventory with the database searched, the corresponding search criteria used, the date when the searches were conducted, and the results will be maintained. A second reviewer will run the searches separately for comparison. The strength of the body of evidence (quality of evidence), the risk of bias and magnitude of effect will be rated and assessed using Grading of Recommendations Assessment, Development and Evaluation (GRADE) [61,62].

*Outcome*. The primary outcome will be adherence to antiretroviral therapy, defined as the proportion of patients meeting the defined adherence criteria. The secondary outcome will be proportion of patients achieving viral suppression, as defined by the study. Outcome (and impact) measures will be reported in terms of changes in the prevalence or reduction in the relative risk. Should there be adequate statistical reporting, a meta-analysis will be considered [63].

#### Data analysis

All adherence interventions or strategies will be described, based on the type of intervention implemented and the setting. The different evaluations methods will then be described in detail by comparing the type of assessments and outcome measures (adherence to ART and viral load). If appropriate, outcome measures will be reported in terms of changes in the prevalence or reduction in the relative risk. Whenever necessary, we will calculate unadjusted risk ratios (RRs) and 95% confidence intervals (CIs) from data provided and present the outcome indicator results in forest plots. Furthermore, we will perform a sensitivity analysis to measure the robustness of our results to the choice of summary statistic and calculated unadjusted risk differences. We will apply a random- effects model to calculate summary RRs and 95% Cl. To test the robustness of the findings, we will re-run the analysis using a fixed effects model. Data will be coded and analysed used STATA version 15.1.

Details for study criteria are presented in Table 1.

### Data management/Data cleaning (phase I-IV)

Data quality scripts for phase I, II, IV will be written in STATA. Data quality checks for phases I, II, and IV (quantitative data) will be done through RedCap (a secure web platform for building and managing research databases). Since TIER.Net will be used as the primary data source for phase I data extraction in the city of Johannesburg region F, where there are data quality issues, the study team will liaise with the facility staff and developmental partners in the region to assist with data clean-up activities. Data quality issues with the ITREMA data (phase II) will be communicated to the ITREMA study quality assurance officer for rectifying. Data quality checks for phase III (qualitative data) will be done in Microsoft Word (before exporting data to NVIVO). All transcripts will be checked for completeness and accuracy against original interviews. Creditability of data analysis will be ensured through triangulation of data sources (i.e., original interviews, field notes, transcripts, medical record).

### Data storage and access (phase I-IV)

Data will be captured and stored electronically, and password protected in the Microsoft Word, Microsoft Excel format and/or RedCap and will only be accessible to an investigator and supervisors only. RedCap access is restricted to only those users who are registered on the system. All data from RedCap, Microsoft Word, Microsoft Excel, STATA, and voice files will be stored in access restricted folders on the Ezintsha server which will only be accessible to an investigator and supervisors. Any paper versions of data will be discarded after use. Data storage and access measures will also comply with data storage and access requirements of the Utrecht University.

### Ethical approvals and consent to participate

We obtained ethical clearance for all the phases of the study from the University of the Witwatersrand Human Research Ethics Committee (clearance certificate number: M190641). Departmental approval was granted by the Johannesburg Health District (DRC Ref: 2019-10-005 and National Health Research Database reference number: GP_201910_031). Written consent for interviews will be obtained from all participants. All participants will be provided with written information about the research, and they will also be verbally informed that their participation is voluntary and that they may withdraw from participation at any time (see S3 Appendix Participant Information sheet).

There are no risks or direct benefits for participating in the study. Participants may benefit by taking part in the study because many people find that it is useful to discuss their experiences, opinions and provide feedback. We believe that the information from this study will help the South African Department of Health better understand and strengthen the ART services provided to patients at large. All participants will be informed of these benefits.

All information discussed during the interview will be kept strictly confidential; at no point will participants’ personal details be disclosed. The consent forms will be kept separately from all other research documents. Only the study team will have access to the information provided by the participants. All dissemination outputs will present de-identified and, where possible, aggregated data.

## Discussion

The overarching aim of this study is to contribute to knowledge that can provide guidance regarding the barriers and facilitators to adherence for first-line and second-line ART patients and different adherence strategies utilized. Using an integrated multilevel socio-ecological framework, this study will focus on determining the influence of the multiple factors that impact on adherence to ART. In line with socio-ecological frameworks [64,65] and propositions, the findings from this study will be discussed under four units of analysis: intrapersonal level, interpersonal level, community level and policy level factors.

### Intrapersonal factors

The client knowledge, attitudes, experiences and perceptions coupled with analysis of the intrapersonal factors influencing adherence to ART play a fundamental role in maintaining adherence [35]. Intrapersonal level factors such as being age, sex, substance abuse, and comorbidities will be discussed, in line with the existing literature [66–69].

### Interpersonal level factors

The trust and communication between patients on ART and treatment supporters is essential in improving and maintaining optimal adherence to treatment [25]. Some studies have reported treatment support as a predictor of adherence [38,70]. In this study, the interpersonal level factors consisting of relationships between patients and family members, friends, intimate partner(s) and healthcare providers will be discussed against previous studies to provide guidance on the role of treatment supporters in strengthening treatment adherence.

### Community level factors

The findings concerning community level factors will consider the importance of patients understanding of social norms, cultural barriers and reduction of poverty, stigma and discrimination against people living with HIV and on ART [71]. Information on poverty, culture, HIV related stigma, and discrimination caused by misconceptions will be discussed in line with existing literature to provide a critical assessment on the role of community level factors on ART adherence.

### Policy level factors

The policy-level factors address awareness and influence of public health policies, guidelines, and standards on patients [72,73]. The South African HIV programme has undergone several changes since its implementation in 2004 [74]. This study will evaluate patient understanding/awareness of ART adherence or HIV treatment related health policies, guidelines, and best practices that are followed by health providers when providing health services. Knowledge of ART medicines or regimens (names of ART drugs), definitions, and clinical functions of viral load (knowledge of threshold for virological failure and suppression) and CD4 cell count (understanding of high or low CD4 cell count) will be discussed as themes in assessing individual’s awareness of existing HIV treatment policies and guidelines. Additionally, we will be able to provide recommendations on required changes at the policy level to improve treatment adherence.

#### Strengths and limitations

To our knowledge this will be the first study using a social-ecological perspective and a convergent parallel mixed method design to report on combination ART in South Africa. Combining quantitative and qualitative data from public health settings, a controlled environment, the patient perspective and evidence from the region will enable us to make recommendations for a comprehensive, acceptable, and appropriate adherence strategy for the country.

The study presents a fewlimitations. The study will be conducted in a total of eight health facilities (seven of over 120 health facilities in one South African metropolitan municipality (urban setting) and one facility in a rural setting). Therefore, findings may not be generalizable to other municipalities and districts in South Africa, or to other country settings. Furthermore, although there are efforts to ensure good quality of data by the South African Department of Health, supporting partners and research staff, secondary data are subject to quality issues, due to data inconsistencies and missing data. Other potential predictors of adherence, such as disclosure, stigma, and self-reported adherence, will be assessed through a standard questionnaire. This may lead to reporting bias (memory and social desirability biases). Policy-level factors might not probe for relevant factors due to how this study is designed (with it focus on the individual’s awareness of health policies and guidelines which is insufficient to fully assess policy-level factors).

In conclusion, our study will demonstrate how an existing socio-ecological conceptual framework can be used as a tool to provide guidance regarding facilitators and barriers to ART adherence. By populating this framework through secondary data analysis, participant interviews of PLHIV who are taking ART and a systematic review comparing adherence intervention strategies for the chronic conditions, this mixed method study will provide evidence on factors affecting treatment adherence at different socio-ecological levels and guide context-specific intervention strategies to improve ART adherence. We believe that the use of our study results to strengthen adherence intervention will subsequently improve health outcomes and decrease the number of patients switching to complex treatment such as second-line and third-line regimens.

### Dissemination

Findings from this research will be submitted for doctoral degree purposes (by thesis). Peer reviewed publications and scientific conference presentations will be developed. Results of the research will be shared with the research participants, donors, and health facilities.

## Supporting information

S1 Appendix Study codebook. (PDF)

S2 Appendix Interview guide. (PDF)

S3 Appendix Participant information sheet. (PDF)

S4 Appendix Systematic review protocol. (PDF)

S5 Appendix PRISMA checklist. (PDF)

S6 Appendix ITREMA questionnaire. (PDF)

## References

[1] Robbins RN, Spector AY, Mellins CA, Remien RH. Optimizing ART Adherence: Update for HIV Treatment and Prevention. Current HIV/AIDS Reports. 2014. doi: 10.1007/s11904-014-0229-5 25304006 PMC4268351

[2] Mannheimer S, Friedland G, Matts J, Child C, Chesney M. The Consistency of Adherence to Antiretroviral Therapy Predicts Biologic Outcomes for Human Immunodeficiency Virus–Infected Persons in Clinical Trials. Clin Infect Dis. 2002; doi: 10.1086/339074 11915001

[3] Pasternak AO, De Bruin M, Jurriaans S, Bakker M, Berkhout B, Prins JM, et al. Modest nonadherence to antiretroviral therapy promotes residual HIV-1 replication in the absence of virological rebound in plasma. J Infect Dis. 2012; doi: 10.1093/infdis/jis502 22927449

[4] Sahay S, Srikanth Reddy K, Dhayarkar S. Optimizing adherence to antiretroviral therapy. Indian Journal of Medical Research. 2011. doi: 10.4103/0971-5916.92629 22310817 PMC3284093

[5] World Health Organization. Section 1: Setting the scene [Internet]. 2003. https://www.who.int/chp/knowledge/publications/adherence_Section1.pdf (accessed 09 February 2020).

[6] World Health Organization. Consolidated guidelines on the use of antiretroviral drugs for treating and preventing HIV infection: recommendations for a public health approach [Internet]. WHO Guidelines. 2013. https://apps.who.int/iris/bitstream/handle/10665/85321/9789241505727_eng.pdf?sequence=1 (accessed 09 February 2020). 24716260

[7] Hertogs K, Bloor S, De Vroey V, Van Den Eynde C, Dehertogh P, Van Cauwenberge A, et al. A novel human immunodeficiency virus type 1 reverse transcriptase mutational pattern confers phenotypic lamivudine resistance in the absence of mutation 184V. Antimicrob Agents Chemother. 2000; doi: 10.1128/AAC.44.3.568-573.2000 10681319 PMC89727

[8] Kozal MJ, Amico KR, Chiarella J, Schreibman T, Cornman D, Fisher W, et al. Antiretroviral resistance and high-risk transmission behavior among HIV-positive patients in clinical care. AIDS. 2004; doi: 10.1097/00002030-200411050-00011 15577652

[9] Achappa B, Madi D, Bhaskaran U, Ramapuram JT, Rao S, Mahalingam S. Adherence to antiretroviral therapy among people living with HIV. N Am J Med Sci. 2013; doi: 10.4103/1947-2714.109196 23626959 PMC3632027

[10] Llibre JM, Pulido F, García F, García Deltoro M, Blanco JL, Delgado R. Genetic barrier to resistance for dolutegravir. AIDS reviews. 2015. 25472016

[11] Cottrell ML, Hadzic T, Kashuba ADM. Clinical pharmacokinetic, pharmacodynamic and drug-interaction profile of the integrase inhibitor dolutegravir. Clinical Pharmacokinetics. 2013. doi: 10.1007/s40262-013-0093-2 23824675 PMC3805712

[12] World Health Organization. Update of recommendations on first-and second-line antiretroviral regimens [Internet]. 2019. https://apps.who.int/iris/bitstream/handle/10665/325892/WHO-CDS-HIV-19.15-eng.pdf (accessed 10 February 2020).

[13] Nachega J B., Marconi V C., van Zyl G U., Gardner E M., Preiser W, Y. Hong S, et al. HIV Treatment Adherence, Drug Resistance, Virologic Failure: Evolving Concepts. Infect Disord—Drug Targets. 2011; doi: 10.2174/187152611795589663 21406048 PMC5072419

[14] Xu L, Munir K, Kanabkaew C, Le Coeur S. Factors influencing antiretroviral treatment suboptimal adherence among perinatally hivinfected adolescents in Thailand. PLoS One. 2017; doi: 10.1371/journal.pone.0172392 28207891 PMC5312953

[15] Onoya D, Nattey C, Budgell E, van den Berg L, Maskew M, Evans D, et al. Predicting the Need for Third-Line Antiretroviral Therapy by Identifying Patients at High Risk for Failing Second-Line Antiretroviral Therapy in South Africa. AIDS Patient Care STDS. 2017; doi: 10.1089/apc.2016.0291 28445088 PMC5446602

[16] Phillips AN, Cambiano V, Nakagawa F, Revill P, Jordan MR, Hallett TB, et al. Cost-effectiveness of public-health policy options in the presence of pretreatment NNRTI drug resistance in sub-Saharan Africa: a modelling study. Lancet HIV. 2018;5(3):e146–54. doi: 10.1016/S2352-3018(17)30190-X 29174084 PMC5843989

[17] Long L, Fox M, Sanne I, Rosen S. The high cost of second-line antiretroviral therapy for HIV/AIDS in South Africa. AIDS. 2010;24(6):915–9. doi: 10.1097/QAD.0b013e3283360976 20042849

[18] Mills EJ, Nachega JB, Bangsberg DR, Singh S, Rachlis B, Wu P, et al. Adherence to HAART: A systematic review of developed and developing nation patient-reported barriers and facilitators. PLoS Medicine. 2006. doi: 10.1371/journal.pmed.0030438 17121449 PMC1637123

[19] Mills EJ, Nachega JB, Buchan I, Orbinski J, Attaran A, Singh S, et al. Adherence to antiretroviral therapy in sub-Saharan Africa and North America: A meta-analysis. Journal of the American Medical Association. 2006. doi: 10.1001/jama.296.6.679 16896111

[20] Posse M, Meheus F, Van Asten H, Van Der Ven A, Baltussen R. Barriers to access to antiretroviral treatment in developing countries: A review. Tropical Medicine and International Health. 2008. doi: 10.1111/j.1365-3156.2008.02091.x 18466183

[21] Simoni JM, Amico KR, Pearson CR, Malow R. Strategies for promoting adherence to antiretroviral therapy: A review of the literature. Current Infectious Disease Reports. 2008. doi: 10.1007/s11908-008-0083-y 18945394 PMC4059830

[22] Berben L, Dobbels F, Engberg S, Hill MN, de Geest S. An Ecological Perspective on Medication Adherence. West J Nurs Res. 2012; doi: 10.1177/0193945911434518 22309989

[23] Haskard Zolnierek KB, Dimatteo MR, Mondala MM, Zhang Z, Martin LR, Messiha AH. Development and Validation of the Physician-Patient Humor Rating Scale. J Health Psychol. 2009; doi: 10.1177/1359105309342288 19858336

[24] Merten S, Kenter E, McKenzie O, Musheke M, Ntalasha H, Martin-Hilber A. Patient-reported barriers and drivers of adherence to antiretrovirals in sub-Saharan Africa: A meta-ethnography. Trop Med Int Heal. 2010; doi: 10.1111/j.1365-3156.2010.02510.x 20586957

[25] Mugavero MJ. Elements of the HIV care continuum: Improving engagement and retention in care. Top Antivir Med. 2016. PMC6148916 27841983

[26] Hirasen K, Evans D, Maskew M, Sanne IM, Shearer K, Govathson C, et al. The right combination—treatment outcomes among HIV-positive patients initiating first-line fixed-dose antiretroviral therapy in a public sector HIV clinic in johannesburg, South Africa. Clin Epidemiol. 2018; doi: 10.2147/CLEP.S145983 29296098 PMC5739109

[27] Nnambalirwa M, Govathson C, Evans D, McNamara L, Maskew M, Nyasulu P. Markers of poor adherence among adults with HIV attending Themba Lethu HIV Clinic, Helen Joseph Hospital, Johannesburg, South Africa. Trans R Soc Trop Med Hyg. 2016;110(12):696–704. doi: 10.1093/trstmh/trx003 28938050 PMC5914359

[28] Orrell C, Cohen K, Mauff K, Bangsberg DR, Maartens G, Wood R. A randomized controlled trial of real-time electronic adherence monitoring with text message dosing reminders in people starting first-line antiretroviral therapy. In: Journal of Acquired Immune Deficiency Syndromes. 2015. p. 495–502. doi: 10.1097/QAI.0000000000000770 26218411

[29] Shubber Z, Mills EJ, Nachega JB, Vreeman R, Freitas M, Bock P, et al. Patient-Reported Barriers to Adherence to Antiretroviral Therapy: A Systematic Review and Meta-Analysis. 2016;1–14. doi: 10.1371/journal.pmed.1002183 27898679 PMC5127502

[30] Azfredrick EC. Using Anderson’s model of health service utilization to examine use of services by adolescent girls in south-eastern Nigeria. Int J Adolesc Youth. 2016; doi: 10.1080/02673843.2015.1124790

[31] ESRC. The Dahlgren–Whitehead rainbow model of health. Economic and Social Research Council. 2018.

[32] Mayberry LS, Osborn CY. Empirical validation of the information-motivation-behavioral skills model of diabetes medication adherence: A framework for intervention. Diabetes Care. 2014;37(5):1246–53. doi: 10.2337/dc13-1828 24598245 PMC3994934

[33] Norton WE, Amico KR, Fisher WA, Shuper PA, Ferrer RA, Cornman DH, et al. Information-motivation-behavioral skills barriers associated with intentional versus unintentional ARV non-adherence behavior among HIV+ patients in clinical care. In: AIDS Care—Psychological and Socio-Medical Aspects of AIDS/HIV. 2010. p. 979–87. doi: 10.1080/09540121003758630 20552469 PMC4286200

[34] Zarani F, Besharat MA, Sadeghian S, Sarami G. The effectiveness of the information-motivation-behavioral skills model in promoting adherence in CABG patients. J Health Psychol. 2010;15(6):828–37. doi: 10.1177/1359105309357092 20453057

[35] Golden SD, Earp JAL. Social Ecological Approaches to Individuals and Their Contexts: Twenty Years of Health Education & Behavior Health Promotion Interventions. Heal Educ Behav. 2012; doi: 10.1177/1090198111418634 22267868

[36] Mayer KH, Mugavero MJ, Amico KR, Horn T, Thompson MA. The state of engagement in HIV care in the United States: From cascade to continuum to control. Clinical Infectious Diseases. 2013. doi: 10.1093/cid/cit420 23797289

[37] Gumede SB, Fischer A, Venter WDF, Lalla-Edward ST. Descriptive analysis of World Health Organization-recommended second-line antiretroviral treatment: A retrospective cohort data analysis. South African Med J. 2019; doi: 10.7196/SAMJ.2019.v109i12.013895 31865953

[38] Gumede SB, Venter WDF, Lalla-Edward ST. Understanding adherence in virally suppressed and unsuppressed human immunodeficiency virus-positive urban patients on second-line antiretroviral treatment. South Afr J HIV Med. 2020; doi: 10.4102/sajhivmed.v21i1.1107 32934834 PMC7479367

[39] Court R, Leisegang R, Stewart A, Sunpath H, Murphy R, Winternheimer P, et al. Short term adherence tool predicts failure on second line protease inhibitor-based antiretroviral therapy: An observational cohort study. BMC Infect Dis. 2014;14(1). doi: 10.1186/s12879-014-0664-3 25472544 PMC4266950

[40] Gross R, Zheng L, La Rosa A, Sun X, Rosenkranz SL, Cardoso SW, et al. Partner-Focused Adherence Intervention for Second-line Antiretroviral Therapy: A Multinational Randomized Trial (ACTG A5234). lancet HIV. 2015;2(1):e12–e19. doi: 10.1016/S2352-3018(14)00007-1 26424232 PMC4313760

[41] Meyer-Rath G, Johnson LF, Pillay Y, Blecher M, Brennan AT, Long L, et al. Changing the South African national antiretroviral therapy guidelines: The role of cost modelling. PLoS One. 2017;12(10):1–15. doi: 10.1371/journal.pone.0186557 29084275 PMC5662079

[42] Marconi VC, Wu B, Hampton J, Ordóñez CE, Johnson BA, Singh D, et al. Early Warning Indicators for First-Line Virologic Failure Independent of Adherence Measures in a South African Urban Clinic. AIDS Patient Care STDS. 2013; doi: 10.1089/apc.2013.0263 24320011 PMC3868291

[43] Rohr JK, Ive P, Horsburgh CR, Berhanu R, Hoffmann CJ, Wood R, et al. Assessing the association between changing NRTIs when initiating second-line ART and treatment outcomes. JAIDS J Acquir Immune Defic Syndr [Internet]. 2017;77(4):1. Available from:. http://insights.ovid.com/crossref?an=00126334-900000000-96787. 10.1097/QAI.0000000000001611 PMC5825249 29206723

[44] Stalter RM, Katayamoyo P, Packer C, Banda H, Chen PL, Mwansa JK, et al. Transitioning to Second-line Antiretroviral Therapy among Adolescents in Copperbelt Province, Zambia: Predictors of Treatment Switching and Adherence to Second-line Regimens. Pediatr Infect Dis J. 2017;36(8):768–73. doi: 10.1097/INF.0000000000001547 28099228

[45] Beehler GP, Funderburk JS, Possemato K, Vair CL. Developing a measure of provider adherence to improve the implementation of behavioral health services in primary care: A Delphi study. Implement Sci. 2013;8(1). doi: 10.1186/1748-5908-8-19 23406425 PMC3640894

[46] Hosseinipour MC, Kumwenda JJ, Weigel R, Brown LB, Mzinganjira D, Mhango B, et al. Second-line treatment in the Malawi antiretroviral programme: high early mortality, but good outcomes in survivors, despite extensive drug resistance at baseline. HIV Med. 2010;11(8):510–8. doi: 10.1111/j.1468-1293.2010.00825.x 20345885 PMC4833877

[47] Lima VD, Bangsberg DR, Harrigan PR, Deeks SG, Yip B, Hogg RS, et al. Risk of viral failure declines with duration of suppression on highly active antiretroviral therapy irrespective of adherence level. J Acquir Immune Defic Syndr. 2010; doi: 10.1097/QAI.0b013e3181f2ac87 20838225 PMC2974791

[48] South African Antiretroviral Treatment Guidelines (Adult) 2015. 2015;(June):6782 (accessed 16 January 2021).

[49] South African National Department of Health. National Consolidated Guidelines for the Prevention of Mother-To-Child Transmission of HIV (PMTCT) and the Managment of HIV in Children, Adolescents and Adults. Dep Heal Repub South Africa. 2015;(April):1–128.

[50] South African National Department of Health. Health indicators update: antiretroviral indicators [Internet]. Pretoria; 2013. https://www.uio.no/studier/emner/matnat/ifi/INF5761/v14/timeplan/artprogramme-overview-from-2004-to-2013_ndoh-2013.pdf (accessed 16 January 2021).

[51] Wensing AMJ. The Intensified Treatment Monitoring Strategy to Prevent Accumulation of Drug Resistance (ITREMA) Trial. https://clinicaltrials.gov/show/NCT03357588. 2017.

[52] Reynolds NR, Sun J, Nagaraja HN, Gifford AL, Wu AW, Chesney MA. Optimizing measurement of self-reported adherence with the ACTG adherence questionnaire: A cross-protocol analysis. J Acquir Immune Defic Syndr. 2007; doi: 10.1097/qai.0b013e318158a44f 18077832

[53] World Health Organization. Consolidated guidelines on the use of antiretroviral drugs for treating and preventing HIV infection: recommendations for a public health approach. WHO Guidel. 2013; https://doi.org/9789241505727 (accessed 16 January 2021). 24716260

[54] Braun V, Clarke V. Thematic Analysis Revised—Final. Qualitative Research in Psychology. 2006. doi: 10.1191/1478088706qp063oa 32100154

[55] Braun V, Clarke V. Using thematic analysis in psychology. Qual Res Psychol. 2006; doi: 10.1191/1478088706qp063oa 32100154

[56] Moher D, Liberati A, Tetzlaff J, Altman DG, Altman D, Antes G, et al. Preferred reporting items for systematic reviews and meta-analyses: The PRISMA statement. PLoS Medicine. 2009. doi: 10.1371/journal.pmed.1000097 21603045 PMC3090117

[57] Gumede S, Venter F WD, de Wit J, Lalla-Edward ST. Adherence strategies and interventions for selected chronic conditions in sub-Saharan Africa: a systematic review and meta-analysis [Internet]. 2020. Available from: https://www.crd.york.ac.uk/prospero/#recordDetails (accessed 22 January 2021).

[58] Adeloye D, Basquill C. Estimating the prevalence and awareness rates of hypertension in Africa: A systematic analysis. PLoS One. 2014; doi: 10.1371/journal.pone.0104300 25090232 PMC4121276

[59] Atun R, Davies JI, Gale EAM, Bärnighausen T, Beran D, Kengne AP, et al. Diabetes in sub-Saharan Africa: from clinical care to health policy. The Lancet Diabetes and Endocrinology. 2017. doi: 10.1016/S2213-8587(17)30181-X 28688818

[60] Remais J V., Zeng G, Li G, Tian L, Engelgau MM. Convergence of non-communicable and infectious diseases in low- and middle-income countries. Int J Epidemiol. 2013; doi: 10.1093/ije/dys135 23064501 PMC3600620

[61] Broazek JL, Akl EA, Compalati E, Kreis J, Terracciano L, Fiocchi A, et al. Grading quality of evidence and strength of recommendations in clinical practice guidelines Part 3 of 3. the GRADE approach to developing recommendations. Allergy: European Journal of Allergy and Clinical Immunology. 2011. doi: 10.1111/j.1398-9995.2010.02530.x 21241318

[62] Guyatt G, Oxman AD, Akl EA, Kunz R, Vist G, Brozek J, et al. GRADE guidelines: 1. Introduction—GRADE evidence profiles and summary of findings tables. J Clin Epidemiol. 2011; doi: 10.1016/j.jclinepi.2010.04.026 21195583

[63] Kanters S, Park JJH, Chan K, Socias ME, Ford N, Forrest JI, et al. Interventions to improve adherence to antiretroviral therapy: a systematic review and network meta-analysis. Lancet HIV. 2017; doi: 10.1016/S2352-3018(16)30206-5 27863996

[64] Dahlberg LL, Krug EG. Violence a global public health problem. Ciencia e Saude Coletiva. 2006. doi: 10.1590/S1413-81232006000200007

[65] Rachlis B, Bakoyannis G, Easterbrook P, Genberg B, Braithwaite RS, Cohen CR, et al. Facility-level factors influencing retention of patients in HIV care in East Africa. PLoS One. 2016; doi: 10.1371/journal.pone.0159994 27509182 PMC4980048

[66] Takarinda KC, Harries AD, Shiraishi RW, Mutasa-Apollo T, Abdul-Quader A, Mugurungi O. Gender-related differences in outcomes and attrition on antiretroviral treatment among an HIV-infected patient cohort in Zimbabwe: 2007–2010. Int J Infect Dis. 2015; doi: 10.1016/j.ijid.2014.11.009 25462184 PMC5072602

[67] Skovdal M, Campbell C, Madanhire C, Mupambireyi Z, Nyamukapa C, Gregson S. Masculinity as a barrier to men’s use of HIV services in Zimbabwe. Global Health. 2011; doi: 10.1186/1744-8603-7-13 21575149 PMC3107786

[68] Fox MP, Berhanu R, Steegen K, Firnhaber C, Ive P, Spencer D, et al. Intensive adherence counselling for HIV-infected individuals failing second-line antiretroviral therapy in Johannesburg, South Africa. Trop Med Int Heal. 2016; doi: 10.1111/tmi.12741 27383454

[69] Nieuwlaat R, Wilczynski N, Navarro T, Hobson N, Jeffery R, Keepanasseril A, et al. Interventions for enhancing medication adherence. Cochrane Database of Systematic Reviews. 2014. doi: 10.1002/14651858.CD000011.pub4 25412402 PMC7263418

[70] Gross R, Zheng L, La Rosa A, Sun X, Rosenkranz SL, Cardoso SW, et al. Partner-based adherence intervention for second-line antiretroviral therapy (ACTG A5234): A multinational randomised trial. Lancet HIV. 2015; doi: 10.1016/S2352-3018(14)00007-1 26424232 PMC4313760

[71] Lifson AR, Demissie W, Tadesse A, Ketema K, May R, Yakob B, et al. HIV/AIDS stigma-associated attitudes in a rural Ethiopian community: Characteristics, correlation with HIV knowledge and other factors, and implications for community intervention. BMC Int Health Hum Rights. 2012; doi: 10.1186/1472-698X-12-6 22553906 PMC3512528

[72] Engler K, Lènàrt A, Lessard D, Toupin I, Lebouché B. Barriers to antiretroviral therapy adherence in developed countries: a qualitative synthesis to develop a conceptual framework for a new patient-reported outcome measure. AIDS Care—Psychol Socio-Medical Asp AIDS/HIV. 2018; doi: 10.1080/09540121.2018.1469725 29719990

[73] Berrouet LM, Machado J, Villegas-Palacio C. Vulnerability of socio—ecological systems: A conceptual Framework. Ecol Indic. 2018; doi: 10.1016/j.ecolind.2017.07.051

[74] Meintjes G, Moorhouse MA, Carmona S, Davies N, Dlamini S, Van Vuuren C, et al. Adult antiretroviral therapy guidelines 2017. South Afr J HIV Med. 2017;18(1):1–24. doi: 10.4102/sajhivmed.v18i1.776 29568644 PMC5843236

