# Supplementary material for: Study protocol: Strengthening understanding of effective adherence strategies for first-line and second-line antiretroviral therapy (ART) in selected rural and urban communities in South Africa
Source: PLoS One. 2021 Dec 21;16(12):e0261107. doi: 10.1371/journal.pone.0261107 (PMC8691643; doi:10.1371/journal.pone.0261107)
Supplement: S1 Appendix — (PDF) [file pone.0261107.s001.pdf]

CODEBOOK FOR ALL STUDY PHASES

Phase I codebook: Secondary data analysis of a large cohort of patients on ART (from TIER.Net database)

**Variables (from TIER.Net)**	**Description and codes**
Patient unique number	Unique identity of a patient. The unique number vary by facilities and may also assist in picking duplicates
Facility	Name of a corresponding facility
Name	Name of the client/patient
Surname	Surname of the client/patient
Date Of Birth	The date of birth of the client/patient
Gender	Male of Female
Age At Pre-ART	This is applicable to patients who attended pre-Art prior to ART initiation and may be seen in older patients (before Universal Test and Treat)
Age At ART Start	This reflects age of the patient when started ART or restarted ART in case of defaulters or LTFU
Current Age	Refers to age of the patient at present (18 years and older)
First Visit Date	The first date the patient was seen in the facility (This date may be the same date as the diagnosis date)
HIV Diagnosis Date	Date the patient was confirmed as HIV positive
ART Start Date	Date of the patient’s first ever experience with triple therapy (date when patient was started on ART for the first time ever) or re-initiated in case of Exp patients
Baseline CD4	First ever CD4 count done after HIV positive diagnosis (sometimes before starting the patient on ART). Before 2016, CD4 count was done after HIV positive status to assess patient's ART elgibility

Regimen At Baseline	First ever regimen issued to the patient when initiated on ART for the first time or when restated on ART (in case of defaulters or LTFU)
Method into ART	Patient has started ART at one facility and has now transferred to another facility
Method Into ART Location	For Transferred/Moved-In patients, facility/location where the patients comes from (referring facility)
Transferred/Moved In Date	If client is a Transferred/Moved-in, Date when the client was transferred in the current clinic
Outcome	"Outcome" reflects active or inactive status of patients (whether the patient is still attending ART services in the facility or not). For active patients, this variable will be blank (however few blanks may also reflects unconfirmed lost to follow-up and this should be verified against Last ART visit date of the patient. In active patients will be coded as either Lost-to-follow up (LTFU), Transferred/Moved out and Died
Outcome Date	For inactive patients (LTFU, Transferred/Moved Out, Died), outcome date reflects date outcome was confirmed. For LTFU and Transferred/Moved Out-it reflects the last time the patient was at the facility
Prior ART	Reflects whether patient has ever taken ART before ART start date. Options listed as NO ART EXP (naïve), PMTCT, Prior ART > 30 days (EXPERIENCE),
Last ART Visit Code	Regimen that was given/issued to the patient at a last visit
Last ART Visit Date	The last visit date when the patient was seen in the facility. It is the date when the Last ART visit code was provided
Last ART CD4 Count	The last or latest CD4 count/result captured on TIER.Net. It is important to highlight that this depends on capturing and data quality i.e. blood results capturing into TIER.Net
Last ART CD4 Date	The date of the Last CD4 Count captured on TIER.Net
Last ART Next Appointment Date	This refers to the next ART appointment given to the patient at last ART visit. This date assists in ensuring that LTFU is calculated accurately
TB Rx Started	Did the client start on TB treatment (Yes,No, Not Sure). This is not at

Last ART VL Count	The last or latest Viral Load result captured on TIER.Net. It is important to highlight that this depends on capturing and data quality i.e. blood results capturing into TIER.Net
Last ART VL Date	The date of the Last Viral Load results captured on TIER.Net.
Second Line Start Date	Second Line Start Date. This is the date a patient was switched from first line to a second line regimen
Last ART Cell Code	Refers to the last regimen issued to the patient or last regimen that was expected to be issued to the patient. This includes the expected visits or appointments where the patients were unable to attend the clinic ("Did Not Attend").
Last ART Cell Date	Refers to the date of the Last ART Cell Code. With the description of a Last ART Cell Code, this might therefore be a future date when looked at against Last ART Visit Date
Last ART Prescription Code	Refers to regimen prescribed to the patient at a last ART visit. Sometimes this is similar to Last ART Visit Code
Last ART Prescription Date	This reflects the date of the last prescription provided to the patient. This may be similar to Last ART Visit Date
TB Status At Last Visit	Reflects TB screening and dianosis at last visit. This includes the following options No Symptoms, Symptoms-With Sputum, Symptoms-No Sputum, On TB Treatment at this facility, On TB treatment at another facility, Not Screened, Screening Status Unknown
Pregnant on ART Start	Was the client/patient pregnant when starting ART
Pap Smear	Reflects whether pap smear was done to the patient (female)
Pap Smear Date	Reflects pap smear test date, if pap smear was done
Last Sub Clinic	Name of the last Sub Clinic. This includes adherence clubs which mostly second line patients will not be allocated to and in case this is captured, data quality may be required
Health Provider At ART Start	Name of the health provider who initiated/started the patient on ART
Pregnancy Status At Last Visit	Refers to pregnancy status in female patients at a last visit (listed as Yes, No or Not Sure). Screening for pregnancy is done at all visits unless the patient is pregnant

ART Restart Date After LTFU	In case the patient defaulted or was declared a LTFU, a date the patient was restarted on ART
Duration On ART (Months)	A period the patient has been on ART since first initiation or re-initation (for experienced patients)

Phase II codebook: Secondary data analysis for the cohort from the ITREMA clinical trial

Question	Code	Numeric or textual	0	1	2	3	4	5	6	7	8	9
id	entry_no	1-600										
part_id	participant_no	ITREMA001ITREMA600										
q4	employment		_		y_self	n_unempl	n_stud	n_ret	n_dis	n_oth	ref	dk
q5	personal_pay	0-100000										
q6a	hh_in_salaries	0-100000										
q6b	hh_in_business	0-100000										
q6c	hh_in_remittance	0-100000										
q6d	hh_in_pension	0-100000										
q6e	hh_in_grants	0-100000										
q6f	hh_in_other	0-100000										
q7a	grant_age		no	yes								
q7b	grant_disability		no	yes								
q7c	grant_child_sup		no	yes								
q7d	grant_care		no	yes								
q7e	grant_foster_child		no	yes								

q7f	grant_veteran		no	yes								
q7g	grant_in_aid		no	yes								
q7h	grant_social		no	yes								
q7i	grant_refused		no	yes								
q7j	NA	NA	NA	NA	NA	NA	NA					

q8a	rela_status		_	married	life_part	half	single	divorced	widowed	multiple	other	
q8b	rela_start	date										
q8c	rela_other	text										
q9	people_under_roo f	0-20										
q10a	hh_partner	0-5										
q10b	hh_child_bio	0-20										
q10c	hh_child_other	0-20										
q10d	hh_parent	0-2										
q10e	hh_in law	0-2										
q10f	hh_sibling	0-20										
q10g	hh_grandparent	0-8										
q10h	hh_other_fam	0-20										

q10i	hh_non_relative	0-20										
q10j	hh_refused		no	yes								
q10k	hh_dk		no	yes								
q11	no_money_food		_	yes	no	dk						
q12	nf_recent		n/a	yes	no	dk						
q13	nf_how_often		n/a	yes	no	dk						
q14	nf_hungry		n/a	yes	no	dk						
q15a	nf_jan		no_n /a	yes								
q15b	nf_feb		no_n /a	yes								
q15c	nf_mar		no_n /a	yes								
q15d	nf_apr		no_n /a	yes								
q15e	nf_may		no_n /a	yes								
q15f	nf_jun		no_n /a	yes								
q15g	nf_jul		no_n /a	yes								
q15h	nf_aug		no_n /a	yes								

q15i	nf_sep		no_n/a	yes								
q15j	nf_oct		no_n/a	yes								
q15k	nf_nov		no_n/a	yes								
q15l	nf_dec		no_n/a	yes								
q15m	nf_dk		no_n/a	yes								
q16	ARV_ability		_	compl_unsure	unsure	sure	compl_sure					
q17	ARV_positive_effect		_	compl_unsure	unsure	sure	compl_sure					
q18	ARV_resistance		_	compl_unsure	unsure	sure	compl_sure					
q19	ss_satisfaction		_	v_dissat	sw_dissat	sw_satis	v_satis					
q20	ss_remember_medication		_	not_at_all	somewhat	a_little	a_lot	n/a				
q21	md_away		_	never	rarely	sometimes	often	n/a				
q22	md_busy		_	never	rarely	sometimes	often	n/a				
q23	md_forgot		_	never	rarely	sometimes	often	n/a				
q24	md_too_many		_	never	rarely	sometimes	often	n/a				
q25	md_side_effect		_	never	rarely	sometimes	often	n/a				

q26	md_privacy		_	never	rarely	someti<br>mes	often	n/a				
q27	md_routine		_	never	rarely	someti<br>mes	often	n/a				
q28	md_harmful		_	never	rarely	someti<br>mes	often	n/a				
q29	md_asleep		_	never	rarely	someti<br>mes	often	n/a				
q30	md_sick		_	never	rarely	someti<br>mes	often	n/a				
q31	md_depression		_	never	rarely	someti<br>mes	often	n/a				
q32	md_specific_time		_	never	rarely	someti<br>mes	often	n/a				
q33	md_empty		_	never	rarely	someti<br>mes	often	n/a				
q34	md_felt_good		_	never	rarely	someti<br>mes	often	n/a				
q35	often_on_time		_	all_time	most_ti<br>me	rarely	never	n/a				
q36	md_days_week		_	every_day	4_to_6	2_to_4	once	less_o<br>nce	never	n/a		
q37	md_last time		_	past_wee<br>k	1_to_2<br>w	2_to_4<br>w	1_to_3<br>m	more_<br>3m	never	n/a		
q38	hh_supp_work		_	no_sup	little_s<br>up	fair_su<br>p	lot_sup					
q39	hh_supp_worries		_	no_sup	little_s<br>up	fair_su<br>p	lot_sup					
q40	hh_supp_leisure		_	no_sup	little_s<br>up	fair_su<br>p	lot_sup					

q41	hh_supp_practical		_	no_sup	little_s up	fair_su p	lot_sup					
q42	hh_supp_personal		_	no_sup	little_s up	fair_su p	lot_sup					
q43	non_hh_supp_wor k		_	no_sup	little_s up	fair_su p	lot_sup					
q44	non_hh_supp_wor ries		_	no_sup	little_s up	fair_su p	lot_sup					
q45	non_hh_supp_leis ure		_	no_sup	little_s up	fair_su p	lot_sup					
q46	non_hh_supp_pra ctical		_	no_sup	little_s up	fair_su p	lot_sup					
q47	non_hh_supp_per sonal		_	no_sup	little_s up	fair_su p	lot_sup					
q48	stress_get_away		_	never	rarely	someti mes	v_often	always				
q49	stress_solve_probl em		_	never	rarely	someti mes	v_often	always				
q50	stress_blame_situ ation		_	never	rarely	someti mes	v_often	always				
q51	stress_treat_food		_	never	rarely	someti mes	v_often	always				
q52	stress_anxious		_	never	rarely	someti mes	v_often	always				
q53	stress_similar_pro blem		_	never	rarely	someti mes	v_often	always				
q54	stress_visit_friend		_	never	rarely	someti mes	v_often	always				
q55	stress_determine_ action		_	never	rarely	someti mes	v_often	always				

q56	stress_buy_me		_	never	rarely	sometimes	v_often	always				
q57	stress_blame_emotional		_	never	rarely	sometimes	v_often	always				
q58	stress_work_understand		_	never	rarely	sometimes	v_often	always				
q59	stress_upset		_	never	rarely	sometimes	v_often	always				
q60	stress_corrective_action		_	never	rarely	sometimes	v_often	always				
q61	stress_blame_knowing		_	never	rarely	sometimes	v_often	always				
q62	stress_special_person		_	never	rarely	sometimes	v_often	always				
q63	stress_think_learn		_	never	rarely	sometimes	v_often	always				
q64	stress_wish_chang e		_	never	rarely	sometimes	v_often	always				
q65	stress_go_food		_	never	rarely	sometimes	v_often	always				
q66	stress_analyze_react		_	never	rarely	sometimes	v_often	always				
q67	stress_inadequaci es		_	never	rarely	sometimes	v_often	always				
q68	stress_phone_friend		_	never	rarely	sometimes	v_often	always				
q69	hcw_depend		_	stro_disagr	disagr	sli_disagr	sli_agree	agree	stro_agree			
q70	hcw_understand		_	stro_disagr	disagr	sli_disagr	sli_agree	agree	stro_agree			

q71	hcw_distrust		_	stro_disagr	disagr	sli_disagr	sli_agree	agree	stro_agree			
q72	hcw_joint_effort		_	stro_disagr	disagr	sli_disagr	sli_agree	agree	stro_agree			
q73	hcw_similar_ideas		_	stro_disagr	disagr	sli_disagr	sli_agree	agree	stro_agree			
q74	hcw_respect		_	stro_disagr	disagr	sli_disagr	sli_agree	agree	stro_agree			
q75	hcw_like		_	stro_disagr	disagr	sli_disagr	sli_agree	agree	stro_agree			
q76	hcw_relationship		_	stro_disagr	disagr	sli_disagr	sli_agree	agree	stro_agree			
q77	hcw_experienced		_	stro_disagr	disagr	sli_disagr	sli_agree	agree	stro_agree			
q78	hcw_likes_me		_	stro_disagr	disagr	sli_disagr	sli_agree	agree	stro_agree			
q79	hcw_distant		_	stro_disagr	disagr	sli_disagr	sli_agree	agree	stro_agree			
q80	CD4_what	text										
q81	ARV_CD4		_	up	down							
q82	VL_what	text										
q83	ARV_VL		_	up	down							
q84	current_med_HIV	text										
q85	take_ARV_bad		_	agree	unsure	disagr						
q86	take_ARV_tired		_	agree	unsure	disagr						

q87	take_ARV_down		_	agree	unsure	disagr						
q88	take_ARV_taste		_	agree	unsure	disagr						
q89	take_ARV_good		_	agree	unsure	disagr						
q90	mental_interest		_	not_at_all	sev_da ys	half_da ys	every_d ay	refuse				
q91	mental_depressed		_	not_at_all	sev_da ys	half_da ys	every_d ay	refuse				
q92	mental_sleep		_	not_at_all	sev_da ys	half_da ys	every_d ay	refuse				
q93	mental_tired		_	not_at_all	sev_da ys	half_da ys	every_d ay	refuse				
q94	mental_appetite		_	not_at_all	sev_da ys	half_da ys	every_d ay	refuse				
q95	mental_me_failur e		_	not_at_all	sev_da ys	half_da ys	every_d ay	refuse				
q96	mental_concentra tion		_	not_at_all	sev_da ys	half_da ys	every_d ay	refuse				
q97	mental_slow_restl ess		_	not_at_all	sev_da ys	half_da ys	every_d ay	refuse				
q98	mental_suicide		_	not_at_all	sev_da ys	half_da ys	every_d ay	refuse				
q99	stigma_dirty		_	stro_disag r	disagr	agree	stro_agr ee					
q100	stigma_cursed		_	stro_disag r	disagr	agree	stro_agr ee					

q101	stigma_trust		_	stro_disag r	disagr	agree	stro_agr ee						
q102	stigma_similar		_	stro_disag r	disagr	agree	stro_agr ee						
q103	stigma_ashamed		_	stro_disag r	disagr	agree	stro_agr ee						
q104	stigma_not_guilty		_	stro_disag r	disagr	agree	stro_agr ee						
q105	stigma_weak		_	stro_disag r	disagr	agree	stro_agr ee						
q106	stigma_safe_childr en		_	stro_disag r	disagr	agree	stro_agr ee						
q107	stigma_restriction s		_	stro_disag r	disagr	agree	stro_agr ee						
q108	stigma_punishmen t		_	stro_disag r	disagr	agree	stro_agr ee						
q109	stigma_isolated		_	stro_disag r	disagr	agree	stro_agr ee						
q110	stigma_allow_wor k		_	stro_disag r	disagr	agree	stro_agr ee						
q111	stigma_friendship		_	stro_disag r	disagr	agree	stro_agr ee						
q112	stigma_family_car e		_	yes	no								
q113	stigma_disclosure _family		_	yes	no								
q114	no_sex_partner_1 2m	0-50											
q115	no_sex_partner_1 m	0-10											

q116a	initial_part1	text										
q116b	initial_part2	text										
q116c	initial_part3	text										
q116d	initial_part4	text										
q116e	initial_part5	text										
q116f	initial_part6	text										
q116g	initial_part7	text										
q116h	initial_part8	text										
q117a	age_part1	0-100										
q117b	age_part2	0-100										
q117c	age_part3	0-100										
q117d	age_part4	0-100										
q117e	age_part5	0-100										
q117f	age_part6	0-100										
q117g	age_part7	0-100										

q117h	age_part8	0-100										
q118a	sex_part1	0-62										
q118b	sex_part2	0-62										
q118c	sex_part3	0-62										
q118d	sex_part4	0-62										
q118e	sex_part5	0-62										
q118f	sex_part6	0-62										
q118g	sex_part7	0-62										
q118h	sex_part8	0-62										
q119a_y	years_part1	0-90										
q119b_y	years_part2	0-90										
q119c_y	years_part3	0-90										
q119d_y	years_part4	0-90										
q119e_y	years_part5	0-90										
q119f_y	years_part6	0-90										
q119g_y	years_part7	0-90										
q119h_y	years_part8	0-90										
q119a_m	months_part1	0-12										

q119b_m	months_part2	0-12										
q119c_m	months_part3	0-12										
q119d_m	months_part4	0-12										
q119e_m	months_part5	0-12										
q119f_m	months_part6	0-12										
q119g_m	months_part7	0-12										
q119h_m	months_part8	0-12										
q119a_d	days_part1	0-31										
q119b_d	days_part2	0-31										
q119c_d	days_part3	0-31										
q119d_d	days_part4	0-31										
q119e_d	days_part5	0-31										
q119f_d	days_part6	0-31										
q119g_d	days_part7	0-31										
q119h_d	days_part8	0-31										

q120a	condom_part1		_	always	half_ti me	rarely	never						
q120b	condom_part2		_	always	half_ti me	rarely	never						
q120c	condom_part3		_	always	half_ti me	rarely	never						
q120d	condom_part4		_	always	half_ti me	rarely	never						
q120e	condom_part5		_	always	half_ti me	rarely	never						
q120f	condom_part6		_	always	half_ti me	rarely	never						
q120g	condom_part7		_	always	half_ti me	rarely	never						
q120h	condom_part8		_	always	half_ti me	rarely	never						
q121	sex_money		_	never	someti mes	often	always						
q122	alcohol		_	never	less_m	monthl y	weekly	daily					
q123	alcohol_six		_	never	less_m	monthl y	weekly	daily					
q124	alcohol_memory		_	never	less_m	monthl y	weekly	daily					
q125a	use_dagga		no	yes									
q125b	use_benzene		no	yes									
q125c	use_mandrax		no	yes									
q125d	use_iv		no	yes									
q125e	use_nyaope		no	yes									
q125f	use_glue		no	yes									

q125g	use_other		no	yes								

Phase III codebook: Participant's understanding of adherence (and their recommendations to improve adherence to medication) (in-depth interviews)

**Themes**	**Guide**	**Codes**
**Participant's life context**	Information about participant's time on ART and mentioning their age, gender, ethnicity and how long you have been on medication. Investigation on their knowledge about ART	**Participant's Life context**<br>**Age**<br>**Gender**<br>**Ethnicity and**<br>**Duration on ART**<br>**Knowledge about ART**
**Treatment history**	Information about the time the particpant was diagnosed with HIV, when they started on treatment, (include the time it took to start treatment). Discussing the time treatment was interrupted or stopped and reasons for interruptions	**Treatment history**<br>**HIV diagnosis**<br>**ART start information**<br>**Treatment interruptions**<br>**Adherence counselling**<br>**Reasons for starting treatment,**<br>**Duration it took to start ART (and reasons for taking longer/shorter),**

	If ever stopped ART for a month or longer, reasons for restarting ARVs again?	**Restarting ART,**<br>**Health complications,**<br>**Self-motivation,**<br>**Adherence counselling,**
		**Support from relative/family member/partner/friends**
**Current use of ART**	Experiences on taking ART medication. What makes participants take their medication. Also discussing current challenges with taking ART correctly or as prescribed	**Factors associated with ART adherence (facilitators and barriers to ART adherence).**<br>**Treatment taking behaviour,**<br>**Motivation to take treatment**<br>**Current challenges or difficulties with taking ART,**<br>**Effects of comorbidities**<br>**Distance to health facilities**<br>**Education on ART adherence**
**Individual level factors (intrapersonal)**	Discussing on the feeling of taking ART/ARVs.	**Regimen related factors**<br>**Effect of size of the pill(s)**<br>**Pill burden**<br>**Dosages**<br>**Frequencies**<br>**Side effects.**
	Discussing the participant's views on substance use (alcohol and drugs) while taking ART or replacing treatment with other non-ART medications or stopping treatment completely?	**Substance use (alcohol and drugs)**<br>**Non-ART medication**<br>**Cultural beliefs and spirituality**<br>**Effects of substance and drug abuse (towards ART adherence) and using non-ART medications, effects**

	Discusing the views on the role of cultural beliefs and spirituality towards ART adherence?	**Influence of religion and cultural beliefs on adherence (both positive and negative)**
	Discussing the role of having employment, financial means or reliable transport to a health facility affects adherence to treatment	**Financial related/economic factors**<br>**Effects of lack of financial support**<br>**Lack of employment**<br>**Poverty**
		**Lack of transportation to a health facility.**
**Relationships level factors (interpersonal)**	Discussing the role of treatment supporters? Or people who help take treatment better? It could be family members, partner, friend or health care worker?	**Treatment support,**<br>**Role of treatment supporters (family members, partner(s), relatives, friends and health care workers.**<br>**Trust**<br>**Confidentiality**<br>**Communication**
**Community-level factors**	Discussing how the community/society feel about people with HIV or on ART?<br><br>The impact of discrimination and stigma towards ART patients?<br><br>Discussig issues around disclosure of HIV status and taking ART<br><br>Discussing veiws about ART services available within the health facility (including adherence services)?	**Societal norms/stigma**<br>**Discrimination and disclosure,**<br>**Societal perceptions and views**<br>**Social norms**<br>**Poverty,**<br>**Disclosure,**<br>**Access and availability of health services within health facilities.**

**Policy level factors (Macrolevel factors or healthcare policy factors)**	Discussing views on the role of ART policies and guidelines towards ART adherence?	**Guidelines and policies,**<br>**Participants have knowledge and an understanding of the role of policies and guidelines**<br>**Guidelines play a role towards ART adherence**
**Treatment taking reminders**	Discussing treatment taking reminders if any	**Treatment taking reminders,**<br>**treatment storage**<br>**Reasons for using the specific reminders and storage.**
**Recommendations for adherence strategies**	Discussing what participants think should be done to improve adherence of patients on ART?	**Recommendations for adherence strategies,**
**Closing**	Discussing any other issues a participant would like to raise regarding ART?	

Phase IV codebook: Systematic review data collection (article extraction form)

	#	Variable / Field Name	Field Label *Field Note*	Field Attributes (Field Type, Validation, Choices, Calculations, etc.)
Instrument: Article extraction form				
	1	record_id	Article number	text
	2	authors	Authors	text
	3	title	Title	text
	4	year_published	Year article published	text
	5	abstract	Abstract	notes
	6	journal	Journal	text
	7	objectives	Objectives	text
	8	population	Study population	text
	9	Study design	Study design	text
	10	sample_size	Sample size	text
	11	adherence_strategy1	Adherence strategy 1	text
	12	adherence_strategy2	Adherence strategy 2	text
	13	adherence_strategy3	Adherence strategy 3	text
	14	adherence_strategy4	Adherence strategy 4	text
	15	adherence_strategy5	Adherence strategy 5	text
	16	comparison_s	Comparison(s)	text
	17	outcome	Outcome	text
	18	comments	Comments	notes
