# Supplementary material for: Study protocol: Strengthening understanding of effective adherence strategies for first-line and second-line antiretroviral therapy (ART) in selected rural and urban communities in South Africa
Source: PLoS One. 2021 Dec 21;16(12):e0261107. doi: 10.1371/journal.pone.0261107 (PMC8691643; doi:10.1371/journal.pone.0261107)
Supplement: S4 Appendix — (PDF) [file pone.0261107.s004.pdf]

Adherence strategies and interventions for selected chronic conditions in sub-Saharan Africa: A systematic review and meta-analysis

Siphamandla B Gumede[1,2*]; John BF de Wit[2]; WD Francois Venter[1]; Samanta T Lalla-Edward[1]; Maaike Noorman[2]

1 Ezintsha, Faculty of Health Sciences, University of the Witwatersrand, Johannesburg, South Africa
2 Department of Interdisciplinary Social Science, Faculty of Social and Behavioural Sciences, Utrecht University

Systematic review conducted by Siphamandla B Gumede

Reviewer: Maaike AJ Noorman (maaikenoorman@live.nl)

Reviewer/Guarantor 1: Samanta T Lalla-Edward (slalla-edward@ezintsha.org)

Reviewer/Guarantor 2: Willem Daniel Francois Venter (fventer@ezintsha.org)

Reviewer/Guarantor 3: John BF de Wit (j.dewit@uu.nl)

Corresponding author* Siphamandla B Gumede

Ezintsha
University of the Witwatersrand,
32 Princess of Wales Terrace, Parktown, Johannesburg, 2193
Email: sgumede@cartafrica.org; sgumede@ezintsha.org

1. Background

Adherence is widely defined as patient's ability to follow a treatment plan and take medications at prescribed times (1–3). Poor adherence to treatment is a limiting factor in the successful health outcomes of numerous health conditions, including HIV, hypertension and diabetes mellitus (DM) (4–6). Globally reports have indicated that up to 50% of treatment for chronic or long term conditions are not taken as recommended by health providers (7–9). In Sub-Saharan Africa, a wide range of barriers to adherence for chronic conditions have been reported, including adverse drug reactions, competing responsibilities, frequencies of treatment intake, tolerability, cost of treatment, food insecurity, stigma, lack of human health resources and social factors (10–12)

In the efforts to address adherence to treatment for the chronic conditions; behavioural and psychological factors, education, integrated care and patient self-management interventions have been explored (12–14). This includes behavioural rehabilitation provided by health providers to patients, integration of pyscho-social support within health programmes, and patient's knowledge about the medication and their overall satisfaction with the treatment (12,15,16). Other studies have recommended telephonic counselling and text messaging or reminders (mobile health), packaging/medication boxes, home visits, drug level monitoring, consistent clinical monitoring of patients (12,17–19). Studies focusing on ART adherence have further emphasized the importance of compliance with standard treatment guidelines (monitoring and reporting of health information (data) to promote appropriate medicine use (10,20–22).

2. Research question

What treatment adherence strategies and interventions for chronic conditions have been tested and implemented in sub-Saharan Africa?

3. Objective

To assess and compare adherence intervention strategies for the chronic conditions of HIV, DM and hypertension which have been tested and implemented in sub-Saharan Africa.

4. Inclusion criteria

a. Population: Patients with selected chronic conditions (HIV, hypertension, DM) in sub-Saharan Africa.

b. Intervention: All interventions listed/described as adherence interventions or strategies for the conditions of HIV, hypertension, DM.

c. Comparisons: Standard of care and other interventions reported on in the review

d. Outcome: The included studies should report any measurement of adherence to chronic conditions – primarily, effects on adherence behaviour and the changes in health outcomes. There is no preferred measurement for reporting; should there be adequate statistical reporting, a meta-analysis will be considered.

e. Setting: All information from sub-Saharan Africa only will be considered for the review.

f. Language: There will be no language restrictions.

g. Date: There will be no date/time restrictions.

h. Publication status: All the documented studies will be considered and included for review. This includes peer reviewed (i.e., papers, manuscripts, and abstracts).

i. Method: The study will be designed and reported according to PRISMA. PICO will be used as a search strategy approach. This study will describe reported adherence programmes and strategies. There will be a focus on behaviour change techniques used or reported changes in process outcomes of adherence programmes and methods of implementation for HIV, hypertension and /or DM.

5. Search strategy and selection procedure

We will search using several electronic databases. These will include PubMed/Medline, Web of Science, Google Scholar, Scopus, and CINAHL. If necessary, we will contact study authors and request more information on individual studies. Citations and bibliographies of records will be reviewed to identify additional relevant material.

The basic search terms included will be:

"Chronic conditions" OR *"hypertension"* OR *"high blood pressure"* OR *"blood pressure"* OR *"arterial hypertension"* OR *"mellitus diabetes type I"* OR *"mellitus diabetes type II"* OR *"Diabetes"* OR *"Sugar"* OR *"HIV"* OR *"Antiretroviral Therapy"* OR *"Antiretroviral Treatment"* OR *"ART"* OR *"ART Programs"* OR *"ART Programmes"* AND *"adherence"* OR *"compliance"* AND *"interventions"* OR *"strategies"* OR *"odds ratio"* OR *"risk ratio"* OR *"evaluation"* OR *"impact"* OR *"effectiveness"* OR *"outcome"* AND *"sub-Saharan Africa"* OR *"sub Saharan Africa"* OR *"sub-Saharan African"* OR *"sub Saharan African"* OR *"Africa" (table 1).*

The search terms will be adjusted to suit the database being searched. An inventory with the database searched, the corresponding search criteria used, the date when the searches were conducted, and the results will be maintained. The principal investigator will do all the searches and the second reviewer will run the searches separately for comparison. The strength of the body of evidence (quality of evidence), the risk of bias and magnitude of effect will be rated and assessed using Grading of Recommendations Assessment, Development and Evaluation (GRADE) (23,24).

6. Data collection and management

A pre-defined data sheet will be developed for data extraction. The tool will include (but not be limited to): reference (author, title), year of publication, setting or location, sample size, intervention description, participants receiving adherence (in case of comparison). The form will be piloted prior to be used for the final searches. The principal investigator will do all the data extraction. A second and third reviewers will conduct a quality control check on the extraction and assist with the full text review of the included material.

Data quality checks will be done through RedCap (a secure web platform for building and managing research databases).

7. Data storage

Data will be captured and stored electronically, and password protected in the Microsoft Excel format and/or RedCap and will only be accessible to an investigator and reviewers only. RedCap access is restricted to only those users who are registered on the system.

8. Analysis

All adherence interventions or strategies will be described, based on the type of intervention implemented and the setting. The different evaluations methods will then be described in detail by comparing the type of assessments and outcome measures. If appropriate, outcome measures will be reported in terms of changes in the prevalence or reduction in the relative risk. Whenever necessary, we will calculate unadjusted risk ratios (RRs) and 95% confidence intervals (CIs) from data provided and present the outcome indicator results in forest plots. Furthermore, we will perform a sensitivity analysis to measure the robustness of our results to the choice of summary statistic and calculated unadjusted risk differences. We will apply a random- effects model to calculate summary RRs and 95% Cl. To test the robustness of the findings, we will re-run the analysis using a fixed effects model. Data will be coded and analysed used STATA version 15.1.

9. Publication

The corresponding author will produce the first draft manuscript which will be commented on by all co-authors. The systematic review will be submitted to a peer-reviewed journal.

10. Duration

The review is expected to take twelve months from protocol development to manuscript submission.

11. References

1. Sahay S, Srikanth Reddy K, Dhayarkar S. Optimizing adherence to antiretroviral therapy. Indian Journal of Medical Research. 2011. https://doi.org/10.4103/0971-5916.92629
2. World Health Organization. Section 1: Setting the scene [Internet]. 2003. Available from: https://www.who.int/chp/knowledge/publications/adherence_Section1.pdf. https://www.who.int/chp/knowledge/publications/adherence_Section1.pdf
3. World Health Organization. Consolidated guidelines on the use of antiretroviral drugs for treating and preventing HIV infection: recommendations for a public health approach [Internet]. WHO Guidelines. 2013. Available from: https://apps.who.int/iris/bitstream/handle/10665/85321/9789241505727_eng.pdf?sequence=1
4. Robbins RN, Spector AY, Mellins CA, Remien RH. Optimizing ART Adherence: Update for HIV Treatment and Prevention. Current HIV/AIDS Reports. 2014. https://doi.org/10.1007/s11904-014-0229-5
5. Mannheimer S, Friedland G, Matts J, Child C, Chesney M. The Consistency of Adherence to Antiretroviral Therapy Predicts Biologic Outcomes for Human Immunodeficiency Virus–Infected Persons in Clinical Trials. Clin Infect Dis. 2002; https://doi.org/10.1086/339074
6. Pasternak AO, De Bruin M, Jurriaans S, Bakker M, Berkhout B, Prins JM, et al. Modest nonadherence to antiretroviral therapy promotes residual HIV-1 replication in the absence of virological rebound in plasma. J Infect Dis. 2012; https://doi.org/10.1093/infdis/jis502
7. Clifford S, Barber N, Elliott R, Hartley E, Horne R. Patient-centred advice is effective

in improving adherence to medicines. Pharm World Sci. 2006; https://doi.org/10.1007/s11096-006-9026-6
8. Pagès-Puigdemont N, Mangues MA, Masip M, Gabriele G, Fernández-Maldonado L, Blancafort S, et al. Patients' Perspective of Medication Adherence in Chronic Conditions: A Qualitative Study. Adv Ther. 2016; https://doi.org/10.1007/s12325-016-0394-6
9. Peterson AM, Takiya L, Finley R. Meta-analysis of trials of interventions to improve medication adherence. Am J Heal Pharm. 2003;
10. Mills EJ, Lester R, Thorlund K, Lorenzi M, Muldoon K, Kanters S, et al. Interventions to promote adherence to antiretroviral therapy in Africa: A network meta-analysis. Lancet HIV. 2014;1(3):e104–11. https://doi.org/10.1016/S2352-3018(14)00003-4
11. Schoffelen AF, Wensing AMJ, Tempelman HA, Geelen SPM, Hoepelman AIM, Barth RE. Sustained Virological Response on Second-Line Antiretroviral Therapy following Virological Failure in HIV-Infected Patients in Rural South Africa. PLoS One. 2013;8(3). https://doi.org/10.1371/journal.pone.0058526
12. Costa E, Giardini A, Savin M, Menditto E, Lehane E, Laosa O, et al. Interventional tools to improve medication adherence: Review of literature. Patient Preference and Adherence. 2015. https://doi.org/10.2147/PPA.S87551
13. Cochrane MG, Bala M V., Downs KE, Mauskopf J, Ben-Joseph RH. Inhaled corticosteroids for asthma therapy: Patient compliance, devices, and inhalation technique. Chest. 2000. https://doi.org/10.1378/chest.117.2.542
14. Hampson SE, Skinner TC, Hart J, Storey L, Gage H, Foxcroft D, et al. Effects of educational and psychosocial interventions for adolescents with diabetes mellitus: A systematic review. Health Technology Assessment. 2001. https://doi.org/10.3310/hta5100
15. Topp SM, Chipukuma JM, Giganti M, Mwango LK, Chiko LM, Tambatamba-Chapula B, et al. Strengthening health systems at facility-level: Feasibility of integrating antiretroviral therapy into primary health care services in Lusaka, Zambia. PLoS One. 2010;5(7). https://doi.org/10.1371/journal.pone.0011522
16. Munro S, Lewin S, Swart T, Volmink J. A review of health behaviour theories : how useful are these for developing interventions to promote long-term medication adherence for TB and HIV / AIDS ? 2007;16:1–16. https://doi.org/10.1186/1471-2458-7-104
17. Williams A, Manias E, Walker R. Interventions to improve medication adherence in people with multiple chronic conditions: A systematic review. Journal of Advanced Nursing. 2008. https://doi.org/10.1111/j.1365-2648.2008.04656.x
18. Piette JD, List J, Rana GK, Townsend W, Striplin D, Heisler M. Mobile health devices as tools for worldwide cardiovascular risk reduction and disease management. Circulation. 2015; https://doi.org/10.1161/CIRCULATIONAHA.114.008723
19. Pinto SL, Gangan N, Gangal N, Shah S. Tools used to improve medication adherence: a systematic review. Value Heal. 2013; https://doi.org/10.1016/j.jval.2013.03.240
20. SIAPS, Gauteng Department of Health. What Are the Reasons for Switching ART Patients to Second- Line Regimen in Public Healthcare Settings in Gauteng ? 2013;(May).
21. Moyo F, Chasela C, Brennan AT, Ebrahim O, Sanne IM, Long L, et al. Treatment outcomes of HIV-positive patients on first-line antiretroviral therapy in private versus public HIV clinics in johannesburg, South Africa. Clin Epidemiol. 2016; https://doi.org/10.2147/CLEP.S93014
22. Evans D, Berhanu R, Moyo F, Nguweneza A, Long L, Fox MP. Can Short-Term Use of Electronic Patient Adherence Monitoring Devices Improve Adherence in Patients

Failing Second-Line Antiretroviral Therapy? Evidence from a Pilot Study in Johannesburg, South Africa. AIDS Behav. 2016;20(11):2717–28. https://doi.org/10.1007/s10461-016-1417-7

23. Broazek JL, Akl EA, Compalati E, Kreis J, Terracciano L, Fiocchi A, et al. Grading quality of evidence and strength of recommendations in clinical practice guidelines Part 3 of 3. the GRADE approach to developing recommendations. Allergy: European Journal of Allergy and Clinical Immunology. 2011. https://doi.org/10.1111/j.1398-9995.2010.02530.x
24. Guyatt G, Oxman AD, Akl EA, Kunz R, Vist G, Brozek J, et al. GRADE guidelines: 1. Introduction - GRADE evidence profiles and summary of findings tables. J Clin Epidemiol. 2011; https://doi.org/10.1016/j.jclinepi.2010.04.026
