# Supplementary material for: Study protocol: Strengthening understanding of effective adherence strategies for first-line and second-line antiretroviral therapy (ART) in selected rural and urban communities in South Africa
Source: PLoS One. 2021 Dec 21;16(12):e0261107. doi: 10.1371/journal.pone.0261107 (PMC8691643; doi:10.1371/journal.pone.0261107)
Supplement: S6 Appendix — (PDF) [file pone.0261107.s006.pdf]

Social Sciences Form

Date of visit	D	D	/	M	M	M	/	Y	Y	Y	Y

ITREMA

V3.0 – 21 Jun 2015

Participant number				Participant initials				

1: DEMOGRAPHICS *(Source: NIDS wave 3)*

**The following questions relate to the participant's demographics**		
(1)	Gender	☐ Female ☐ Male
(2)	Date of Birth	DD/MMM/YYYY:
(3)	Education	☐ None<br>☐ Grade ___ (specify 1-12)<br>☐ Technikon / College<br>☐ University
	Home language	______________________________

2: EMPLOYMENT & INCOME COMPOSITION *(Source: NIDS wave 3)*

**The following questions relate to the participant's personal employment and income.**		
**Q4**<br>(5)	Are you currently employed?	☐ Yes, employed<br>☐ Yes, self employed<br>☐ No, unemployed →Go to **Q6**<br>☐ No, student →Go to **Q6**<br>☐ No, retired →Go to **Q6**<br>☐ No, disabled →Go to **Q6**<br>☐ No, other →Go to **Q6**<br>Specify: ______________________<br><br>☐ Refused →Go to **Q6**<br>☐ Don't know →Go to **Q6**
**Q5**<br>(8)	What was your personal take home pay last month? (if no income received write 0)	__________<br><br>☐ Refused<br>☐ Don't know
**The following questions relate to the participant's household employment and income.**		
**Q6**<br>(9, 10)	What are the sources of income for this household?<br><br>(Select all applicable. For each field, fill out the total income for the whole household)	☐ Salaries/wages/commission (amount received per month:_______)<br>☐ Income from a business (amount received per month:_______)<br>☐ Remittance (money received from people living elsewhere) (amount received per month:_______)<br>☐ Pensions (amount received per month:_______)<br>☐ Other income sources e.g. rental income, interest (amount received per month:_______)<br>No income → **Go to Q11**
**Q7**<br>(11)	Do you receive any of the listed financial support? (select all that apply)	☐ Old-age grant (60-74, R1200; 75+, R1220)<br>☐ Disability grant (<60, R1200)<br>○ Permanent disability<br>○ Temporary disablity<br>○ Don't know

Date of visit	D	D	/	M	M	M	/	Y	Y	Y	Y

Participant number				Participant initials				

		☐ Child support grant (0-16, R280)<br>☐ Care dependency grant (0-17, R1200)<br>☐ Foster child grant (<22, R770)<br>☐ War veterans grant (60+, R1220)<br>☐ Grant-in-aid (R250 and should have another grant)<br>☐ Social relief of distress<br>☐ Refused<br>☐ Don't know

3: HOUSEHOLD COMPOSITION & PARTNERSHIP STATUS *(Source: SAHANES)*

**The following questions relate to the participant's household composition and partnership status**		
**Q8** (4)	What is your partnership status?<br><br>(Select the answer that best fits the current situation. Please only select one)	☐ Married (since:__________ year)<br>☐ Life partner (since: __________ year)<br>☐ Living together >50% of the time (since: ___________ year)<br>☐ Single (since: __________ year)<br>☐ Divorced (since: __________ year)<br>☐ Widowed (since: __________ year)<br>☐ Multiple partners (since: __________ year)<br>Other (specify: ____________________________________)
**Q9** (14)	How many persons live under your roof?	____________ Number
**Q10** (15)	How do these people relate to you?	☐ Partner (number ____________)<br>☐ Biological children (number ____________)<br>☐ Other children (number ____________)<br>☐ Parent(s) (number ____________)<br>☐ Parent(s) in law (number ____________)<br>☐ Brothers/sisters (number ____________)<br>☐ Grandparents (number ____________)<br>☐ Other family (number _____________)<br>☐ Non-relatives (number ____________)<br>☐ Refused<br>☐ Don't know

**Date of visit**	D	D	/	M	M	M	/	Y	Y	Y	Y

**Participant number**			

**Participant initials**				

4: FOOD SECURITY *(Source: SAHANES)*

**The following questions relate to the availability of food in the participant's household**		
**Q11** (52)	Did your household run out of money to buy food during the past 12 months?	☐ Yes<br>☐ No → **Go to Q16**<br>☐ Don't know
**Q12** (53)	Has it happened in the past 30 days?	☐ Yes<br>☐ No → **Go to Q14**<br>☐ Don't know
**Q13** (54)	Has it happened 5 or more days in the past 30 days?	☐ Yes<br>☐ No<br>☐ Don't know
**Q14** (55)	In the past 12 months, were there times when members of your household went hungry because there was not enough food in the house to eat?	☐ Yes<br>☐ No<br>☐ Don't know
**Q15** (56)	Which were the months (in the last 12 months) in which you experienced a lack of food or money such that one or more members of your household had to go hungry?	☐ January<br>☐ February<br>☐ March<br>☐ April<br>☐ March<br>☐ April<br>☐ May<br>☐ June<br>☐ July<br>☐ August<br>☐ September<br>☐ October<br>☐ November<br>☐ December<br>☐ Don't know

Date of visit	D	D	/	M	M	M	/	Y	Y	Y	Y

Participant number				Participant initials				

5: ADHERENCE *(Source: ACTG)*

**The following questions are about how the participant feels about starting to take ARV's.**					
"These questions are about how people feel about starting to take ARV's. How sure are you that…"		*Not at All Sure*	*Somewhat Sure*	*Very Sure*	*Extremely Sure*
**Q16** *(94)*	*You will be able to take all or most of the medication as directed?*	☐	☐	☐	☐
**Q17** *(95)*	*The medication will have a positive effect on your health?*	☐	☐	☐	☐
**Q18** *(96)*	*If you do not take this medication exactly as instructed, the HIV in your body will become resistant to HIV medication?*	☐	☐	☐	☐

"The following questions ask about your social support…"		
**Q19** *(97)*	*In general, how satisfied are you with the overall support you get from your friends and family members?*	☐ *Very Dissatisfied*<br>☐ *Somewhat Dissatisfied*<br>☐ *Somewhat Satisfied*<br>☐ *Very Satisfied*
**Q20** *(98)*	*To what extent do your friends or family members help you remember to take your medication?*	☐ *Not At All*<br>☐ *A Little*<br>☐ *Somewhat*<br>☐ *A Lot*<br>☐ *Not Applicable*

People may miss their medication for various reasons. Here is a list of possible reasons why the patient may have missed taking any medications **<u>he/she was already taking</u>** within the **<u>past month</u>**.					
"In the past month, how often have you missed taking your medication because you…"		*Never*	*Rarely*	*Sometimes*	*Often*
**Q21** *(99)*	*Were away from home?*	☐	☐	☐	☐
**Q22** *(100)*	*Were busy with other things?*	☐	☐	☐	☐
**Q23** *(101)*	*Simply forgot?*	☐	☐	☐	☐
**Q24** *(102)*	*Had too many pills to take?*	☐	☐	☐	☐
**Q25** *(103)*	*Wanted to avoid side effects?*	☐	☐	☐	☐
**Q26** *(104)*	*Did not want other notice you taking medication?*	☐	☐	☐	☐
**Q27** *(105)*	*Had a change in daily Routine?*	☐	☐	☐	☐
**Q28** *(106)*	*Felt like drug was toxic/harmful?*	☐	☐	☐	☐
**Q29** *(107)*	*Fell asleep/slept through dose time?*	☐	☐	☐	☐

**Date of visit**	D D / M M M / Y Y Y Y
**Participant number**	
**Participant initials**	

**Q30** *(108)*	*Felt sick or ill?*	☐	☐	☐	☐
**Q31** *(109)*	*Felt depressed/overwhelmed?*	☐	☐	☐	☐
**Q32** *(110)*	*Had problem taking pills at a specified times (with meals, on empty stomach etc)?*	☐	☐	☐	☐
**Q33** *(111)*	*Ran out of pills?*	☐	☐	☐	☐
**Q34** *(112)*	*Felt good?*	☐	☐	☐	☐

**Q35** *(113)*	*How often do you have difficulty in taking your medication on time? By "on time" we mean no more than two hours before or after the time your doctor instructed you to take it. (Check one box)*	☐ *All of the time*<br>☐ *Most of the time*<br>☐ *Rarely*<br>☐ *Never*
**Q36**	*On average how many days per week would you say that you missed at least one dose of your medication? (Check one box)*	☐ *Every day*<br>☐ *4 – 6 days per week*<br>☐ *2 – 3 days per week*<br>☐ *Once a week*<br>☐ *Less than once a week*<br>☐ *Never*
**Q37**	*When was the last time you missed taking any of your medications? (Check one box)*	☐ *within the past **week***<br>☐ *1 – 2 **week**s ago*<br>☐ *2 – 4 **week**s ago*<br>☐ *1 – 3 **months** ago*<br>☐ *More than 3 **months** ago*<br>☐ ***Never***

Date of visit	D	D	/	M	M	M	/	Y	Y	Y	Y

Participant number				Participant initials				

6: ACTUAL SUPPORT FROM HOUSEHOLD MEMBERS *(Source: NKPS)*

**This segment is about support the participant receives from household members**					
	"To what extent do persons in your household support you…"	*No support*	*A little support*	*A fair amount of support*	*A lot of support*
**Q38**	*In decisions about work or education*	1	2	3	4
**Q39**	*When you have worries or health problems*	**1**	**2**	**3**	**4**
**Q40**	*In your leisure time activities and social contacts*	**1**	**2**	**3**	**4**
**Q41**	*With all kinds of practical things you need to do*	**1**	**2**	**3**	**4**
**Q42**	*In personal matters that are on your mind*	**1**	**2**	**3**	**4**

7: ACTUAL FAMILY SUPPORT *(Source: NKPS)*

**This segment is about support the participant receives from family members who do not live in the household**					
	"To what extent do family members who do not live in your household support you…"	*No support*	*A little support*	*A fair amount of support*	*A lot of support*
**Q43**	*In decisions about work or education*	1	2	3	4
**Q44**	*When you have worries or health problems*	**1**	**2**	**3**	**4**
**Q45**	*In your leisure time activities and social contacts*	**1**	**2**	**3**	**4**
**Q46**	*With all kinds of practical things you need to do*	**1**	**2**	**3**	**4**
**Q47**	*In personal matters that are on your mind*	**1**	**2**	**3**	**4**

Date of visit D D / M M M / Y Y Y Y

Participant number

Participant initials

8: COPING ABILITIES *(Source: CISS-21)*

This segment is about how the participant reacts to various difficult, stressful or upsetting situations. Every possible reaction needs to be scaled from "never" to "always".						
	"During a difficult, stressful or upsetting situation, how often do you do the following…"	*Never*	*Rarely*	*Sometimes*	*Very often*	*Always*
**Q48**	*Take some time off and get away from the situation*	*1*	*2*	*3*	*4*	*5*
**Q49**	*Focus on the problem and see how I can solve it*	**1**	**2**	**3**	**4**	**5**
**Q50**	*Blame myself for having gotten into this situation*	**1**	**2**	**3**	**4**	**5**
**Q51**	*Treat myself to a favourite food or snack*	**1**	**2**	**3**	**4**	**5**
**Q52**	*Feel anxious about not being able to cope*	**1**	**2**	**3**	**4**	**5**
**Q53**	*Think about how I solved similar problems*	**1**	**2**	**3**	**4**	**5**
**Q54**	*Visit a friend*	**1**	**2**	**3**	**4**	**5**
**Q55**	*Determine a course of action and follow it*	**1**	**2**	**3**	**4**	**5**
**Q56**	*Buy myself something*	**1**	**2**	**3**	**4**	**5**
**Q57**	*Blame myself for being too emotional on the situation*	**1**	**2**	**3**	**4**	**5**
**Q58**	*Work to understand the situation*	**1**	**2**	**3**	**4**	**5**
**Q59**	*Become very upset*	**1**	**2**	**3**	**4**	**5**
**Q60**	*Take corrective action immediately*	**1**	**2**	**3**	**4**	**5**
**Q61**	*Blame myself for not knowing what to do*	**1**	**2**	**3**	**4**	**5**
**Q62**	*Spend time with a special person*	**1**	**2**	**3**	**4**	**5**
**Q63**	*Think about the event and learn from my mistakes*	**1**	**2**	**3**	**4**	**5**
**Q64**	*Wish that I could change what had happened or how I felt*	**1**	**2**	**3**	**4**	**5**
**Q65**	*Go out for a snack or meal*	**1**	**2**	**3**	**4**	**5**
**Q66**	*Analyze the problem before reacting*	**1**	**2**	**3**	**4**	**5**
**Q67**	*Focus on my general inadequacies*	**1**	**2**	**3**	**4**	**5**
**Q68**	*Phone a friend*	**1**	**2**	**3**	**4**	**5**

Date of visit	D	D	/	M	M	M	/	Y	Y	Y	Y

Participant number				Participant initials				

9: CAREGIVER TRUST *(Source: The Helping Alliance – HAQ2)*

**The following statements are about the patient's relationship with the caregiver at Ndlovu Medical Centre. Please indicate to what extent the patient agrees or disagrees with the statement.**							
"To what extent do you agree or disagree with the following statements"		*Strongly disagree*	*Disagree*	*Slightly disagree*	*Slightly agree*	*Agree*	*Strongly Agree*
**Q69**	*I feel I can depend upon the clinician.*	☐	☐	☐	☐	☐	☐
**Q70**	*I feel the clinician understands me.*	☐	☐	☐	☐	☐	☐
**Q71**	*At times I distrust the clinician's judgment.*	☐	☐	☐	☐	☐	☐
**Q72**	*I feel I am working together with the clinician in a joint effort.*	☐	☐	☐	☐	☐	☐
**Q73**	*I believe we have similar ideas about the nature of my problems.*	☐	☐	☐	☐	☐	☐
**Q74**	*I generally respect the clinician's views about me.*	☐	☐	☐	☐	☐	☐
**Q75**	*I like the clinician as a person.*	☐	☐	☐	☐	☐	☐
**Q76**	*A good relationship has formed with my clinician.*	☐	☐	☐	☐	☐	☐
**Q77**	*The clinician appears to be experienced in helping people.*	☐	☐	☐	☐	☐	☐
**Q78**	*I believe the clinician likes me as a person.*	☐	☐	☐	☐	☐	☐
**Q79**	*At times the clinician seems distant.*	☐	☐	☐	☐	☐	☐

10: HEALTH LITERACY*(Source: BEHKA-HIV)*

**The following questions are used to check the participant's knowlegde of HIV. Let patients answer on their own, without giving hints.**				
"We would like to know if patients are familiar with two HIV terms: "CD4-count" and "viral load". Would you mind if I ask you a few questions abouth that?"				
**Q80**	*What is a CD4-count?*			
**Q81**	*Is the goal of ARV's to make the CD4-count go UP or DOWN?*	☐ *UP*	☐ *DOWN*	
**Q82**	*What is a viral load?*			
**Q83**	*Is the goal of ARV's to make the viral load go UP or DOWN?*	☐ *UP*	☐ *DOWN*	
**Q84**	*What medicines are you currently taking to treat HIV?*			
"Please tell me if you agree, are not sure, or disagree with the following statements."				
**Q85**	*I don't take my ARV's when they make me feel bad.*	☐ *Agree*	☐ *Unsure*	☐ *Disagree*

Date of visit D D / M M M / Y Y Y Y

Participant number ☐☐☐

Participant initials ☐☐☐☐

**Q86**	*I don't take my ARV's when I am too tired.*	☐ *Agree*	☐ *Unsure*	☐ *Disagree*
**Q87**	*I don't take my ARV's when I am feeling down or low.*	☐ *Agree*	☐ *Unsure*	☐ *Disagree*
**Q88**	*I don't take my ARV's because it tastes bad.*	☐ *Agree*	☐ *Unsure*	☐ *Disagree*
**Q89**	*I don't take my ARV's when I feel good.*	☐ *Agree*	☐ *Unsure*	☐ *Disagree*

11: MENTAL HEALTH *(Source: PHQ-9)*

**The following questions ask about symptoms. For each symptom, the symptom frequency must be given.**						
"Over the last 2 weeks, how often have you been bothered by any of the following problems?"		*Not at all*	*Several days*	*More than half the days*	*Nearly every day*	*Refuse*
**Q90** *(18)*	*Little interest or pleasure in doing things*	☐	☐	☐	☐	☐
**Q91** *(19)*	*Feeling down, depressed, or hopeless*	☐	☐	☐	☐	☐
**Q92** *(20)*	*Trouble falling or staying asleep, or sleeping too much*	☐	☐	☐	☐	☐
**Q93** *(21)*	*Feeling tired or having little energy*	☐	☐	☐	☐	☐
**Q94** *(22)*	*Poor appetite or overeating*	☐	☐	☐	☐	☐
**Q95** *(23)*	*Feeling bad about yourself — or that you are a failure or have let yourself or your family down*	☐	☐	☐	☐	☐
**Q96** *(24)*	*Trouble concentrating on things, such as reading the newspaper or watching television*	☐	☐	☐	☐	☐
**Q97** *(25)*	*Moving or speaking so slowly that other people could have noticed? Or the opposite — being so fidgety or restless that you have been moving around a lot more than usual*	☐	☐	☐	☐	☐
**Q98** *(26)*	*Thoughts that you would be better off dead or of hurting yourself in some way*	☐	☐	☐	☐	☐

Date of visit D D / M M M / Y Y Y Y

Participant number ☐☐☐ **Participant initials** ☐☐☐☐

12: STIGMATIZATION *(Source: AIDS-related stigma scale)*

**The following questions relate to stigma. Ask the participant if he/she agrees with these statements**					
"Do you agree with the following statements?"		*Strongly disagree*	*Disagree*	*Agree*	*Strongly Agree*
**Q99** *(34)*	*People who have AIDS are dirty*	☐	☐	☐	☐
**Q100** *(35)*	*People who have AIDS are cursed*	☐	☐	☐	☐
**Q101** *(36)*	*People who have AIDS cannot be trusted*	☐	☐	☐	☐
**Q102***(37)*	*People who have AIDS are like everybody else*	☐	☐	☐	☐
**Q103** *(38)*	*People who have AIDS should be ashamed*	☐	☐	☐	☐
**Q104***(39)*	*People who have AIDS have nothing to feel guilty about*	☐	☐	☐	☐
**Q105** *(40)*	*Most people become HIV positive by being weak or foolish*	☐	☐	☐	☐
**Q106** *(41)*	*It is safe for people who have AIDS to work with children*	☐	☐	☐	☐
**Q107** *(42)*	*People who have AIDS must expect restrictions on their freedom*	☐	☐	☐	☐
**Q108** *(43)*	*A person with AIDS must have done something wrong and deserves to be punished*	☐	☐	☐	☐
**Q109** *(44)*	*People who have HIV should be isolated*	☐	☐	☐	☐
**Q110** *(45)*	*People who have AIDS should not be allowed to work*	☐	☐	☐	☐
**Q111**	*I do not want to be friends with someone who has HIV/AIDS*	☐	☐	☐	☐
**Q112***(46)*	*Would you care for a family member with HIV/AIDS?*	☐ *Yes*<br>☐ *No*			
**Q113** *(47)*	*Would you mind if people knew if your family member had HIV/AIDS*	☐ *Yes*<br>☐ *No*			

Date of visit	D	D	/	M	M	M	/	Y	Y	Y	Y

Participant number				Participant initials				

13: SEXUAL PARTNERS & TRANSACTIONAL SEX *(Source: UNAIDS sexual networks)*

**These questions are about the patient's sexual partners in the last 12 months**									
**Q114** *(48)*	*How many sexual partners have you had in the last 12 months?*	________ *Number*							
**Q115** *(50)*	*How many sexual partners do you currently have?*	________ *Number*							
"I would like to ask you about people you had sex with ***in the last 12 months****. Please only give this information if you are comfortable giving it. We will not disclose any of the information you give us."*									
**Q116**	*What are the partners initials?*	1 ______	2 ______	3 ______	4 ______	5 ______	6 ______	7 ______	8 ______
**Q117** *(49)*	*What is the partners age?*								
**Q118**	*How many times in the last month did you have sex with this person?*	______	______	______	______	______	______	______	______
**Q118**	*How long has the relationship been going on?* *Years* *Months* *Days*	______ ______ ______	______ ______ ______	______ ______ ______	______ ______ ______	______ ______ ______	______ ______ ______	______ ______ ______	______ ______ ______
**Q120**	*How often do you have sex with this partner without a condom?* **Always 1** **Often 2** **Half of the time 3** **Rarely 4** **Never 5**	**1 2 3 4 5**	**1 2 3 4 5**	**1 2 3 4 5**	**1 2 3 4 5**	**1 2 3 4 5**	**1 2 3 4 5**	**1 2 3 4 5**	**1 2 3 4 5**
**Q121** *(51)*	*Do you ever have sex in exchange for money or goods?*	☐ *Never* ☐ *Sometimes* ☐ *Often* ☐ *Always*							

Date of visit	D	D	/	M	M	M	/	Y	Y	Y	Y

Participant number				Participant initials				

14: SUBSTANCE ABUSE *(Source: CHAMP study questionnaire)*

**These questions ask about the participant's use of drug and alcohol**						
		Never	***Less than monthly***	***Monthly***	***Weekly***	***Daily / almost daily***
Q121	*How often do you have a drink containing alcohol?*					
Q122	*Do you have six or more drinks on one occasion?*					
Q123	*In the past year were you unable to remember what happened the night before because of your drinking?*					

Q124	*Have you ever used any of the following …*<br><br>*You may choose more than one answer*	*Dagga*	
		Benzene or petrol	
		Mandrax	
		Injected drugs (drugs that you inject)	
		Nyaope	
		Sniff glue	
		Other drug	
