# Supplementary material for: Study protocol: Strengthening understanding of effective adherence strategies for first-line and second-line antiretroviral therapy (ART) in selected rural and urban communities in South Africa
Source: PLoS One. 2021 Dec 21;16(12):e0261107. doi: 10.1371/journal.pone.0261107 (PMC8691643; doi:10.1371/journal.pone.0261107)
Supplement: Attachment — Submitted filename: Gumede et al_Response to reviewers.pdf [file pone.0261107.s007.pdf]

Ezintsha

27 October 2021

Dear PLOS One editorial team,

Thank you for the opportunity to improve the manuscript. Please find enclosed the revised manuscript "**Strengthening understanding of effective adherence strategies for first-line and second-line antiretroviral therapy (ART) in selected rural and urban communities in South Africa**" as well as a point-by-point answer to each of the comments requesting revision.

The revisions were completed within the short turnaround time requirement, and we hope the revised manuscript will better suit the required standard by the journal and respond to the reviewers' feedback. We also declare that there are no ethical or legal restrictions on sharing a de-identified data set.

These revisions have been approved by all authors.

Sincerely,
Mr. Siphamandla Gumede (on behalf of all authors)

Ezintsha, a sub-division of Wits RHI

Central Office: 5th Floor, Block D, Sunnyside Office Park, 32 Princess of Wales Terrace, Parktown 2193, Johannesburg, South Africa www.ezintsha.org

WITS RHI

INVESTIGATE INNOVATE ADVANCE

Note: Not explicit in the protocol - this is a protocol for a doctoral degree by publication, therefore it is being presented as multiple stand-alone studies which will lead to different publications – as per the requirements of the university postgraduate review committee.

**Journal Requirements**	**Authors' responses and action plan**
We note that you have provided funding information that is currently declared in your Funding Statement. However, funding information should not appear in the Acknowledgments section or other areas of your manuscript. We will only publish funding information present in the Funding Statement section of the online submission form.<br>Please remove any funding-related text from the manuscript and let us know how you would like to update your Funding Statement. Currently, your Funding Statement reads as follows:	We have removed any funding-related text from the manuscript.<br><br>The funding statement should remain unchanged, as:<br><br>*The principal investigator/SBG is funded by the Utrecht University (UU), Department of Interdisciplinary Social Science, Netherlands (https://www.uu.nl/en/organisation/faculty-of-social-and-behavioural-sciences), and by the Carnegie Corporation of New York (https://www.carnegie.org/) (Grant No--B 8606.R02), Sida (https://www.sida.se/en) (Grant No:54100113), the DELTAS Africa Initiative (https://www.aasciences.africa/aesa/programmes/developing-excellence-leadership-training-and-science-africa-deltas-africa#) (Grant No: 107768/Z/15/Z) and Deutscher Akademischer Austauschdienst (DAAD) (https://www.daad.de/en/). The DELTAS Africa Initiative is an independent funding scheme of the African Academy of Sciences (AAS)'s Alliance for Accelerating Excellence in Science in Africa (AESA) and supported by the New Partnership for Africa's Development Planning and Coordinating Agency (NEPAD Agency) with funding from the Wellcome Trust (UK) and the UK government.*<br><br>*WDFV and STL-E are funded by the National Heart, Lung, And Blood Institute of the National Institutes of Health (https://www.nhlbi.nih.gov/) under Award Number UG3HL156388 and Fogarty International Centre. The content is solely the responsibility of the authors and does not necessarily represent the official views of the National Institutes of Health.*<br><br>*The funders had and will not have a role in study design, data collection and analysis, decision to publish, or preparation of the manuscript.*

1. Please ensure that your manuscript meets PLOS ONE's style requirements, including those for file naming. The PLOS ONE style templates can be found at: https://journals.plos.org/plosone/s/file?id=wjVg/PLOSOne_formatting_sample_main_body.pdf and https://journals.plos.org/plosone/s/file?id=ba62/PLOSOne_formatting_sample_title_authors_affiliations.pdf.	Noted with thanks. We have checked the template and style requirements and believe that all is in order
2. We note that you have indicated that data from this study are available upon request. PLOS only allows data to be available upon request if there are legal or ethical restrictions on sharing data publicly. For more information on unacceptable data access restrictions, please see http://journals.plos.org/plosone/s/data-availability#loc-unacceptable-data-access-restrictions.	
a) If there are ethical or legal restrictions on sharing a de-identified data set, please explain them in detail (e.g., data contain potentially sensitive information, data are owned by a third-party organization, etc.) and who has imposed them (e.g., an ethics committee). Please also provide contact information for a data access committee, ethics committee, or other institutional body to which data requests may be sent.<br>b) If there are no restrictions, please upload the minimal anonymized data set necessary to replicate your study findings as either Supporting Information files or to a stable, public repository and provide us with the relevant URLs, DOIs, or accession numbers. For a list of acceptable repositories, please see http://journals.plos.org/plosone/s/data-availability#loc-recommended-repositories.	There are no ethical or legal restrictions on sharing a de-identified dataset and we can share the dataset, however since this is a protocol submission which does not contain results and only partial raw data may be available (this is an ongoing study) we have opted not to include data. All datasets from this study will be published with their relevant manuscripts. We are not definite on which journals those would be and cannot be certain that the journal will decline to review the manuscript since the data was already published.<br>If still a requirement and granted, we will contact PLOS One and could share the de-identified datasets with PLOS One once each relevant phase has been published even if it is with different journals. At this stage we are not entirely certain that sharing datasets and do not want to compromise those publications – most especially as this study is for degree purposes.
3. Please include captions for your Supporting Information files at the end of your manuscript, and update any in-text citations to match accordingly. Please see our Supporting Information guidelines for more information: http://journals.plos.org/plosone/s/supporting-information.	Captions have been added at the end of manuscript (after the references). The in-text references have been updated.

4. Please review your reference list to ensure that it is complete and correct. If you have cited papers that have been retracted, please include the rationale for doing so in the manuscript text, or remove these references and replace them with relevant current references. Any changes to the reference list should be mentioned in the rebuttal letter that accompanies your revised manuscript. If you need to cite a retracted article, indicate the article's retracted status in the References list and also include a citation and full reference for the retraction notice.	Reference list has been reviewed and amended accordingly. According to our knowledge, we have not cited any retracted papers.

Reviewer #1

**Comments**	**Authors' responses and action plan (please note that line numbers are in line with the document with TRACK CHANGES). Please refer in text to check all the corrections to comments (instead of using line numbers only)**
Given the complexity of the study, it would be helpful to explain why each component is needed and how results from the different components will come together to address the overarching study aims. It would be especially helpful if the researchers could provide an overarching central hypothesis and hypotheses for each of the research questions, and then comment on how the components will come together to address the overarching aims of the study.	We have included an explanation on why each study phase is needed and how it will come together to address the overarching aim (line 198-200). An overarching hypothesis has been included in the Rationale section of the manuscript (line 155-156). Separate hypotheses have been included with each research question.<br><br>It is important to also highlight that this is doctoral protocol to be submitted by publication and each study phase will support the doctoral requirements. The protocol has largely been formatted to the requirements/satisfaction of the postgraduate review committee.
First, the framework and the analysis do not include access-related enablers such as distance to clinic/pharmacy, wait time in clinic/pharmacy, frequency of clinical visits and ART re-supply visits, etc. These have been shown to influence ART medication possession and adherence. How will these be accounted for in the framework and analysis?	Access related enablers/barriers will be collected in the qualitative component and reported on in phase 3.<br><br>Noting your points however, the adapted socio-ecological framework which we are using has been appropriately edited. Most of these access factors will be collected from patients themselves. As such it speaks to intrapersonal/individual level factors. Aside from demographics, it is largely related to previous experiences.

Second, medication reimbursement policies are included in the framework, but will any study participants be exposed to requirements for any form of payment for ART? How will this be evaluated, given that ART medications are available free-of-cost in public sector clinics in South Africa?	We agree with this comment. This was initially intended to address financial means to access care. We have now added access under the individual level and removed the financial reimbursement component in the framework factors to try to probe for the financial related factors to accessing health services and overall adherence.
Third, the way in which the researchers plan to elicit policy-level factors seems to be probing not the policy-level factors themselves (which have a direct effect on adherence) but rather an individual's understanding of health policies and guidelines. These seem to be two different things and it is not clear that one can be substituted for the other. For example, a patient may not be familiar with what the current policies are, or the names of ART medications, but they may still be affected by policies, for example, by being prescribed those medications, whether or not they know their names.	We acknowledge that the policy level factors may not probed fully due to the focus of the study (individual level factors). We will be mindful of this point during the interviews and will probe accordingly without leaving out crucial points. In addition, we have added this as a study limitation in the discussion (line 598-601):<br><br>*Policy-level factors might not probe for relevant factors due to how this study is designed (with it focus on the individual's understanding of health policies and guidelines which is insufficient to fully assess policy-level factors).*
Finally, in the Introduction, the following claim is unsubstantiated and contrasts with the earlier discussion of numerous extant frameworks for understanding adherence. "Unfortunately, most adherence studies are not guided by any conceptual framework and subsequently are unable to draw adherence strategies from conceptual frameworks of any kind."	Thank you for raising this point. The sentence has been edited as follows (line 124-126):<br><br>*Unfortunately, there are no quantitative studies of combination ART in South Africa (and even sub-Saharan Africa more generally) that have used a social-ecological perspective or approach.*
Appendices are thorough and detailed. Phase 2 codebook is missing a data dictionary/glossary for the variable abbreviations.	The dictionary/glossary for the phase 2 study is not available from the ITREMA team as they do not consider it a requirement for their study procedures (beyond what has already been included in this submission). We have however added a detailed phase 2 study questionnaire as a supplementary document to provide more information on the variable abbreviations.
Abstract: open parenthesis before "(using" is never closed.	This has been corrected in the manuscript (line 11)
Introduction:<br><br>a) "adherences" – I'm not sure this can be plural<br>b) "However, individual-level factors are only able to report a limited portion of the variability in non-adherence." – here the subject 'individual-level factors' is not doing the verb 'report.'	Corrections made as follows:<br><br>a) Adherences to adherence (line 49)<br>b) However, using individual-level factors one is only able to report a limited proportion of the variability in non-adherence (22) (line 55-56)<br>c) Portion to proportion (line 56)

c) Also 'portion' → proportion?<br>"Good interpersonal relationships between patients and care givers or treatment supporters including healthcare providers, an intimate partner, family members, and friends have been reported as a predictor for good<br>d) adherence." – should make 'a predictor' plural<br>e) "intrapersonal, interpersonal" – word repeated	d) A predictor to predictors (line 59)<br>e) No change made - "intrapersonal, interpersonal" – no word repeated

Reviewer #2

**Authors' comments**	**Authors' responses and action plan (please note that line numbers are in line with the document with TRACK CHANGES). Please refer in text to check all the corrections to comments (instead of using line numbers only)**
Despite the ambitious plan of conducting a four phases study and the use of a predefined conceptual framework, I am not sure that the proposed methodology allows to reach the expectations. In addition there is little explanation about how mixed methods will be applied.	As mentioned earlier this is a protocol for a doctoral degree by publication and has been written up to meet the requirements of the university review committee. This study is a convergent parallel mixed methods design for the whole study (and not components) where each study component will use different methods (quantitative, qualitative, and systematic review) but will converge at the point of discussion and recommendations for the intervention design/next steps for adherence in people using ART in South Africa.<br><br>A sentence to explain the convergence has been added to the Study Design section directly under Materials and Methods (line 198-200).<br><br>In line with both your and reviewer one's comments, we have made some changes to the framework and how It will adapt some aspects of the data collection. This combined with what has already been completed (analysis for phases 1 and 2, partial data collection for phase 3 and the systematic review) in the 6 months from submission of this protocol manuscript for review we (my supervisors and I) are confident that I will successfully complete the proposed study.
The research gap is not well documented by an in-depth analysis of the abundant literature available on the subject and falls short of building a strong justification (rational) to the study.	The Research Gap section has been edited to include the following (line 121-140):

	In South Africa specifically, there is lack of reported knowledge about the effectiveness and impact of strategies currently employed to promote adherence in people living with HIV (PLHIV) who are taking ART (particularly young patients, males, and those experiencing severe treatment related side effects) (37,38). Unfortunately, there are no quantitative studies of combination ART in South Africa (and even sub-Saharan Africa more generally) that have used a social-ecological perspective or approach. This is despite the understanding that patients on ART require comprehensive adherence strategies and strict viral load monitoring (39,40).<br>*Until 2017, there were minimal related treatment adherence strategies (routine blood monitoring, targeted adherence counselling, and adherence clubs (41–43)) employed by the South African government (41). However, effectiveness of these interventions have not been widely reported on. Additionally, the patients' perspectives seem to be given little consideration in the adherence intervention development and implementation. It is therefore important to assess effectiveness of existing strategies from the perspectives of PLHIV taking ART in order to recommend and develop relevant and acceptable strategies to patients (44,45). This is evidenced in a study conducted in Malawi which highlighted how critical it is for health programmers to prioritize adherence view points from patients as part of strengthening the clinical monitoring strategy used (46).*
Research questions: it is not clear to me the difference between the first and the second question if not for the setting (urban vs rural) and the used dataset (cohort vs RCT). Or is it that the first question uses virological failure and the second adherence as main outcome? but in data analysis the selected outcomes are the same.	These two phases primarily differ by setting (urban vs. rural) and are standalone as they will be two separate publications when the study is completed.<br><br>However as this is secondary data, there are indeed other differences. The research questions have been amended to highlight the differences.
Material and methods:<br>Phase 1: the possible sample size for this study is very interesting, but the type of dataset is very limited. This should be discussed as well as how missing data will be managed (missing data are very frequent in routinely collected data).	Records with the missing data will be excluded in the final analysis. This will be after conducting all necessary data quality checks, verifications, and triangulations with other data sources (line 241-243).<br><br>In terms of the type of analysis and time frame: Analysis will include survival analysis which will consider different entry time points into the ART

Another important point to discuss is the time frame of the analysis. Will all patients be evaluated at the 12-month follow up? adherence being dynamic, possible barriers and facilitators will be very different for someone after 1 year of treatment or after 17 years despite similar characteristics at baseline.<br><br>How survival bias will be managed? (the less adherent may have died over time) How this point will be managed?<br><br>Some of the control variables seem not available in the list provided (codebook)	program. Subsequently, patients will not be simply grouped all together but will be followed up in a 12-month interval when measuring the outcomes (virological failure and lost to follow up) (line 264-267).<br><br>For an example:<br>Patients initiated in 2004 will have 16 years of follow up<br>Patients initiated in 2018 will only have 2 years of follow up<br><br>In order to prevent survival bias, we have ensured that all patient outcomes are included in the analysis. This includes patients with recorded outcome as "dead". In addition, patients without any outcome who had missed their appointment by over 90 days were regarded as lost to follow up and were also included in the final analysis.<br><br>Control variables have been corrected as per the codebook
Phase 2: this study is a secondary analysis of data collected during an RCT. The trial should be presented to understand the population involved and the type of intervention evaluated to allow the reader to understand if the study can answer the question. Nowhere is defined how adherence is measured and the codebook provided is unintelligible to the non initiated to the database. Again the outcome variables are ill defined and do not include adherence (see research question)	We have added more information (and reference 51) on the ITREMA RCT, and the type of intervention evaluated in the study design section for this phase (line 277-285).<br><br>A section defining the outcomes for this study which include self-reported adherence, pill count and viral load has been added to the data collection section for phase 2 (line 303-316).<br><br>Lastly, we have added an ITREMA questionnaire as a supplementary document (S7) to help the reader understand in detail the type of variables that were collected in the RCT
Phase 3: I think this may be the most fruitful phase of the study as it stands on primarily collected data. The other comment on this phase is that I feel that even if interviewed patients can provide their perspectives on health system and policy factors, the research to be multilevel should include perspectives from the other "levels" included in the framework, being them service providers or policy makers.	We are of the opinion, based on our experience on the ground and involvement in technical working groups that some of the information collected in this study can reach programmers, policies, and guidelines. However, we do note your point and will include this as a study limitation.<br><br>Healthcare provider and policy-maker perspectives have been considered in another ongoing study we

	are involved in which uses the community based participatory approach.
Phase 4: I do not understand why the study protocol for this phase is provided in supplementary material instead of in the main body of the protocol. I am not experienced in protocols for systematic review and meta-analysis, but my impression is that neither in the main protocol nor in the one provided in the supplementary material the study methodology is very clear. What is the difference between interventions and strategies for the authors? will they compare single adherence support activities or categories of intervention (Cognitive behavioral therapy? incentives? Behavioral skills training? etc) Will they consider only clinical trials? how will they manage the quality of the study selected? How will they deal with the different measurement of outcomes? I feel that there is already a lot of work done on adherence and if the methodology of review is not rigorous also in terms of choice of outcome and way of measuring, the study will contribute only to more confusion. on this point I advise to include in the bibliography Lancet HIV 2017; 4: e31–40	It Is provided as a supplementary document instead of the main body of the protocol primarily for readability, compliance with journal formatting requirements, and to also to minimize making the content confusing. This systematic review was also registered as is (*Gumede S, WD Venter F, de Wit J, Lalla-Edward ST. Adherence strategies and interventions for selected chronic conditions in sub-Saharan Africa: a systematic review and meta-analysis [Internet]. 2020. Available from: https://www.crd.york.ac.uk/prospero/#recordDetailshttps://doi.org/https://www.crd.york.ac.uk/prospero/#recordDetails).*<br>The systematic review protocol was guided by the PICO tool.<br><br>Strategies are taken to refer to the plans reported on which could comprise multiple interventions. Interventions are taken to refer to individual components/ actions/ models employed.<br><br>In addition to what has already been included in the protocol, we will describe the interventions and strategies separately and we will compare interventions of the same categories and between categories. What would eventually be doable would be dependent on the data available/reported/extracted.<br><br>The recommended reference has been included in the manuscript (line 471).
Concerning availability of data after the study, very little is discussed; with a generic "data can be made available on reasonable request". I understand that more and more it is requested that data are made public on specific repository. This should be discussed.	As this is a protocol manuscript, we have not included and do not have data to completely include with the paper. It is intended to publish the datasets with the relevant manuscripts as the studies are published. The data availability statement has been edited to reflect this. At this stage we are not entirely certain that sharing datasets for a protocol paper will not compromise the future individual publications.
Concerning ethical aspects: in this paragraph it is usually requested to discuss benefits and risks for participants and the measures taken to safeguard confidentiality and privacy of participants. The obtained ethical clearance concerns all the phases of the protocol?	The Ethical approvals and consent to participate section has been revised to read as below (line 513-535):<br><br>*We obtained ethical clearance for all the phases of the study from the University of the Witwatersrand Human Research Ethics Committee (clearance*

	certificate number: M190641). Departmental approval was granted by the Johannesburg Health District (DRC Ref: 2019-10-005 and National Health Research Database reference number: GP_201910_031). Written consent for interviews will be obtained from all participants (see S3 Appendix_informed consent form). All participants will be provided with written information about the research, and they will also be verbally informed that their participation is voluntary and that they may withdraw from participation at any time (see S4 Appendix_Information sheet).<br>*There are no risks or direct benefits for participating in the study. Participants may benefit by taking part in the study because many people find that it is useful to discuss their experiences, opinions and provide feedback. We believe that the information from this study will help the South African Department of Health better understand and strengthen the ART services provided to patients at large. All participants will be informed of these benefits.*<br><br>*All information discussed during the interview will be kept strictly confidential; at no point will participants' personal details be disclosed. The consent forms will be kept separately from all other research documents. Only the study team will have access to the information provided by the participants. All dissemination outputs will present de-identified and, where possible, aggregated data.*
Discussion: readers are expecting in this chapter the discussion of the strengths and limits of the study, how the study fits with the multilevel socio-ecological framework, and how the parallel mixed method design will enrich interpretation of the data. In fact what is missing is the mixing of the methods: the protocol as presented it is more a multi-studies protocol, not indicating how the mixing of methods will be done.	As a convergent parallel study, this is a mixed method for the entire study not one phase, we will be using routine contextual data, patient perspective data and systematic review data that will guide the discussions, recommendations, and the design of future adherence interventions for implementation with people receiving ART in South Africa<br><br>An explanation of how the data will converge is included in the Study Design section immediately under Materials and Methods (line 198-200).<br><br>A Strengths and Limitation section has been added to the Discussion (line 583-601).

Minor comments:<br><br>a) update the paragraph about WHO first line recommandations to include dolutegravir<br>b) check that all abbreviations are given first in extent<br>c) it is useful to define a single primary outcome to make the statistics, with the other endpoints being secondary<br>d) it is useful to give the definitions used for each variable and its categories: in the supplementary material it is not very clear for the study phase 2	All minor comments have been corrected and addressed in the manuscript as follows:<br><br>a) updated as follows (line 43-45):<br>*WHO recommends the use of the HIV drug dolutegravir (DTG) as the preferred first-line and second-line ART treatment regimens for all populations due to its formidable resistance barrier and improved tolerability (10–12).*<br><br>b) Abbreviations have been defined in full the first time and used as abbreviations throughout thereafter.<br>c) Outcomes have been defined for each study phase.<br>d) As mentioned, the only definitions and categories that were made available by the RCT team have been included in this codebook. We did not predefine any thresholds for those variables and the RCT team did not predefine them because the results could not be predicted. We have added a study questionnaire as a supplementary document to provide more information on the variables and the categories.
