# Supplementary material for: Study protocol: Strengthening understanding of effective adherence strategies for first-line and second-line antiretroviral therapy (ART) in selected rural and urban communities in South Africa
Source: PLoS One. 2021 Dec 21;16(12):e0261107. doi: 10.1371/journal.pone.0261107 (PMC8691643; doi:10.1371/journal.pone.0261107)
Supplement: Attachment — Submitted filename: Gumede et al_Response to reviewers.pdf [file pone.0261107.s008.pdf]

Ezintsha

22 November 2021

Dear PLOS One editorial team,

Thank you for the opportunity to improve the manuscript. Please find enclosed the revised manuscript "**Strengthening understanding of effective adherence strategies for first-line and second-line antiretroviral therapy (ART) in selected rural and urban communities in South Africa**" as well as a point-by-point answer to each of the comments requesting revision.

We hope the revised manuscript will better suit the required standard by the journal and respond to the reviewers' feedback.

These revisions have been approved by all authors.

Sincerely,
Mr. Siphamandla Gumede (on behalf of all authors)

Ezintsha, a sub-division of Wits RHI
Central Office: 5th Floor, Block D, Sunnyside Office Park, 32 Princess of Wales Terrace, Parktown 2193, Johannesburg, South Africa www.ezintsha.org

WITS RHI

INVESTIGATE INNOVATE ADVANCE

Journal Requirements	Authors' responses and action plan
Please review your reference list to ensure that it is complete and correct. If you have cited papers that have been retracted, please include the rationale for doing so in the manuscript text, or remove these references and replace them with relevant current references. Any changes to the reference list should be mentioned in the rebuttal letter that accompanies your revised manuscript. If you need to cite a retracted article, indicate the article's retracted status in the References list and also include a citation and full reference for the retraction notice	We have reviewed the reference list and made minor edits (links and date accessed) on the following references:<br><br>*Reference 5, 6, 12, 43, 50, 53, 57*<br><br>According to our knowledge, we have not cited any retracted articles/papers.

Reviewer #1

Comments	Authors' responses and action plan
Now that I understand this paper is for a student's degree requirement, I recommend that the student iterate the hypotheses with their academic advisor in order to choose hypotheses based on gaps in the field. The hypothesis that "PLHIV who have optimum adherence to ART and remain in care will have better treatment and clinical outcomes" does not appear to be a gap in the field because it is well-known than HIV care and adherence to ART improves clinical outcomes and indeed saves lives of PLHIV.	We have revised the hypotheses. These hypotheses are derived from the existing knowledge and theories regarding ART adherence, access to care, and treatment behaviours and perspectives. They are based on the study objectives and relative to gaps in the study settings/context. We have tried to address your comment while still staying within the content of our regulatory approved protocol.<br><br>***Overarching hypothesis:***<br><br>A socio-ecological framework combined with multi-model data collection will identify the barriers and facilitators to ART adherence and retention in care for PLHIV needed to strengthen adherence interventions.<br><br>**Research Question (RQ) 1 Hypothesis:**<br>Demographic and clinical characteristics are associated with virological failure and being LTFU

	in patients on ART from a complex, and highly mobile urban communities.<br><br>**RQ 2 Hypothesis:** Individual and community level factors are associated with self-reported non-adherence, suboptimal pill count, and virological failure in patients on ART in the rural setting.<br><br>**RQ 3 Hypothesis:** In South African communities, treatment taking behaviour, perceptions of adherence, and recommendations for adherence interventions differ between PLHIV who are virally unsuppressed and those who are virally suppressed.<br><br>**RQ 4 Hypothesis:** There are no differences in the effectiveness of adherence interventions implemented in sub-Saharan Africa for HIV, hypertension, and DM.
The critique regarding inappropriate methods for evaluating policy-level factors was not adequately addressed. I recommend changing the study aims/framing to be evaluating comprehension/awareness of policy, rather than evaluating the effect of policy on patient outcomes, which this study is not currently designed to do.	Thank you for your comment. We have changed the framing where relevant to "evaluating comprehension/awareness of policies and/or guidelines"<br>Relevant changes have been made in the following lines in manuscript (in the draft with track changes)<br>Fig. 1<br>Line 60<br>Line 104-105<br>Line 337<br>Line 381-382<br>Line 563-575<br>Line 594
Typo: "asherence"	Thank you for picking this up. Word "asherence" has been removed together with the initial hypothesis 3, since it has been revised (line 180-185)

Reviewer #2

Authors' comments	Authors' responses and action plan

The authors have considered reviewers' comments and amended the protocol consequently. The overarching hypothesis is not of the most in need of confirmation, but the final results will be of interest for the global knowledge on adherence and for the local authorities for policy making.<br>As the authors state at different sections of their answer, the protocol is for a doctoral thesis and have to comply with different requirements. I congratulate the authors for their major endeavor in realizing all these studies	Thank you for this feedback. We have also taken note of your comment regarding the overarching hypothesis, and we have reworked on it to make sure it is aligned with the study aim, objective, and the study gap.
